# Regulation of NF-κB by the p105-ABIN2-TPL2 complex and RelAp43 during rabies virus infection

**DOI:** 10.1371/journal.ppat.1006697

**Published:** 2017-10-30

**Authors:** Benoit Besson, Florian Sonthonnax, Magalie Duchateau, Youcef Ben Khalifa, Florence Larrous, Hyeju Eun, Véronique Hourdel, Mariette Matondo, Julia Chamot-Rooke, Regis Grailhe, Hervé Bourhy

**Affiliations:** 1 Unité Dynamique des Lyssavirus et Adaptation à l’Hôte, Paris, France; 2 Université Paris Diderot, Sorbonne Paris Cité, Cellule Pasteur, Paris, France; 3 Unité de Spectrométrie de Masse Structurale et Protéomique, Plateforme Protéomique, CNRS USR 2000 Spectrométrie de masse pour la biologie, Paris, France; 4 Technology Development Platform, 16, Daewangpangyo-ro 712 beon-gil, Bundang-gu, Seongnam-si, Gyeonggi-do, Rep. of Korea; Thomas Jefferson University, UNITED STATES

## Abstract

At the crossroad between the NF-κB and the MAPK pathways, the ternary complex composed of p105, ABIN2 and TPL2 is essential for the host cell response to pathogens. The matrix protein (M) of field isolates of rabies virus was previously shown to disturb the signaling induced by RelAp43, a NF-κB protein close to RelA/p65. Here, we investigated how the M protein disturbs the NF-κB pathway in a RelAp43-dependant manner and the potential involvement of the ternary complex in this mechanism. Using a tandem affinity purification coupled with mass spectrometry approach, we show that RelAp43 interacts with the p105-ABIN2-TPL2 complex and we observe a strong perturbation of this complex in presence of M protein. M protein interaction with RelAp43 is associated with a wide disturbance of NF-κB signaling, involving a modulation of IκBα-, IκBβ-, and IκBε-RelAp43 interaction and a favored interaction of RelAp43 with the non-canonical pathway (RelB and p100/p52). Monitoring the interactions between host and viral proteins using protein-fragment complementation assay and bioluminescent resonance energy transfer, we further show that RelAp43 is associated to the p105-ABIN2-TPL2 complex as RelAp43-p105 interaction stabilizes the formation of a complex with ABIN2 and TPL2. Interestingly, the M protein interacts not only with RelAp43 but also with TPL2 and ABIN2. Upon interaction with this complex, M protein promotes the release of ABIN2, which ultimately favors the production of RelAp43-p50 NF-κB dimers. The use of recombinant rabies viruses further indicates that this mechanism leads to the control of *IFN*β, *TNF* and *CXCL2* expression during the infection and a high pathogenicity profile in rabies virus infected mice. All together, our results demonstrate the important role of RelAp43 and M protein in the regulation of NF-κB signaling.

## Author summary

Rabies virus is a recurring zoonosis responsible for about 60,000 deaths per year. A key feature of rabies virus is its stealth, allowing it to spread within the host and escape the immune response. To do so, rabies virus developed several mechanisms, including a thorough interference with cell signaling pathways. Here, we focused our attention on the molecular aspects of rabies virus escape to the NF-κB pathway through the interaction between the M protein and the NF-κB protein RelAp43. Monitoring close range interactions, we found that RelAp43 plays an important role in the stabilization of the p105-ABIN2-TPL2 complex, which is essential in the regulation of both NF-κB and MAPK pathways, and we brought a new insight on the dynamics within the host protein complex. These results were confirmed in living cells and in mice. Overall, our data suggest that rabies virus interference with the p105-ABIN2-TPL2 complex is a cornerstone of its stealth strategy to escape the immune response.

## Introduction

Depending on specific stimuli, the transcription factors of the NF-κB family are major regulators of cellular physiology. They regulate apoptosis, cell survival, proliferation and immune response, which requires each step of the cell signaling cascade to be highly regulated [1]. The NF-κB family is constituted of 3 proteins with a transactivation domain (TAD): RelA (p65), cRel and RelB and 3 proteins lacking a TAD: p105/p50 (NF-κB1), p100/p52 (NF-κB2) and RelAp43, a sixth member of the NF-κB family and splicing variant of RelA [2]. All these proteins share a Rel Homology Domain (RHD) involved in dimerization, DNA- and IκB binding. The NF-κB pathway is divided in a canonical pathway, which mostly relies on RelA-p105/p50 dimers, and a non-canonical pathway involving RelB-p100/p52 dimers. Upon activation of the cascade, the IκB kinase (IKK) complex is phosphorylated and induces the phosphorylation of IκB proteins, such as IκBα or IκBβ, leading to their ubiquitination and degradation by the proteasome. Similarly, p100 and p105, acting both as precursors and inhibitors, are phosphorylated and cleaved into maturated p52 and p50 upon IKK activation. Therefore, NF-κB dimers are released from their inhibitors and free to translocate into the nucleus to regulate the expression of their target genes.

Interestingly, a pool of p105 is also forming a complex with the A20-binding inhibitor of NF-κB activation 2 (ABIN2) and the Tumor progression locus 2 (TPL2). Both ABIN2 and TPL2 interact with the C-terminal (C-ter) half of p105 and not with p50 [3]. TPL2, stabilized and inhibited by p105, is a key MAP3K involved in the activation of the immune response mediated by the MAPK pathway through downstream kinases MEK1/2 and ERK1/2. ABIN2 is essential to maintain steady-state TPL2 levels, so that in ABIN2 depleted cells, the activation of downstream ERK is reduced under stimulation [4]. TPL2 induces the NF-κB pathway without prior stimulation in a p105-dependant manner [5], increasing the production of p50 and its translocation in the nucleus together with RelA, thereby activating NF-κB-responsive reporter genes. Interestingly, TPL2 phosphorylates p105 at different sites compared to IKK and is autophosphorylated in unstimulated cells [6]. It has been suggested that TPL2 promotes the full processing of p105 by the proteasome, releasing independent NF-κB dimers while maintaining the overall rate of p50 production. Indeed, TPL2 could modulate the NF-κB pathway while being associated to a restricted pool of p105 forming the core of high-molecular-weight regulatory complexes [3,7]. Finally, upon IKK activation, p105 is phosphorylated by the regulatory IKKγ/NEMO subunit and ABIN2 is released from the complex together with TPL2, which once liberated, can activate the ERK/MAPK pathway. This ternary complex then establishes an intersection step between both NF-κB and MAPK pathways, notably involved in the regulation of *IFNß*, *CXCL2*, *TNF*, *CCL5* or *CXCL10* [8,9], constituting a target of choice for immune escape by viruses such as *Lyssavirus*, the agents of rabies.

Lyssaviruses are already known to interfere with several signaling pathways [10]. One of the 5 proteins coded by the lyssavirus genome, the phosphoprotein (P) inhibits both the IRFs [11] and the JAK-STAT pathways [12] through a mechanism conserved across the *Lyssavirus* genus [13]. Another viral protein, the matrix protein (M), is a small pleiotropic protein forming oligomers and involved in various functions such as structural organization of the viral particle or potentially in the formation of viral inclusions [14,15]. First involved in the dysregulation of cell homeostasis and induction of cell death [16–18], the M protein has been also shown to be associated with the inhibition of the NF-κB pathway through an interaction with RelAp43 [2,19]. Interestingly, only the M protein of field rabies virus (RABV) isolates such as the Thailand strain (M_Tha_) was shown to interact with RelAp43, compared to attenuated viral strains such as the vaccinal PV or SAD virus (M_SAD_). Recently, 4 residues of the M protein (position 77, 100, 104, 110) were shown to be involved in the interaction of M_Tha_ with the C-ter part of RelAp43 and linked to the control of the expression of RelAp43-dependant genes [19].

Here we further characterize the modulation of the NF-κB network mediated by RelAp43 and the role of M_Tha_ in the formation of RelAp43-p50 dimers through its interaction with a complex formed by RelAp43, NF-κB1 (p105/p50), ABIN2 and TPL2 (RNAT). Interestingly, although M_Tha_, M_SAD_ and M_Th4M_ (a defective mutant) are interacting with the complex, we can distinguish several profiles of interaction. All M proteins can interact with TPL2, but only M_Tha_ has a strong interaction for both RelAp43 and ABIN2. Thereby only the M_Tha_ protein and Tha virus are able to affect NF-κB signaling leading to the control of the host response to the infection. Taken together, our data bring new insight in the NF-κB pathway and particularly on the p105-ABIN2-TPL2 complex, shedding light on the role of the matrix protein on cell signaling and on viral immune evasion.

## Results

### Mapping of RelAp43 interactors highlights an important modulation of the NF-κB network by M_Tha_ and identification of ABIN2 as a major target of M_Tha_

Using RelAp43 as bait in a tandem affinity purification (TAP) assay associated with a label-free quantitative proteomics approach, we identified 329 proteins and 49 of them were shown to be significantly interacting–directly or not—with RelAp43 (S1 Fig). For 38 of these proteins, the interaction with RelAp43 was observed to be modulated (5 decreased and 33 increased) in the presence of M_Tha_. The network of RelAp43 interactome was build based on hits identified after RelAp43 purification (S2 Fig), revealing highly intra- and inter-connected clusters of protein. Besides the NF-κB pathway, RelAp43 appears to form complexes with proteins involved in gene expression regulation (mRNA splicing, nucleoplasm), protein expression regulation (translation, ubiquitin-proteasome) or the regulation of protein localization (cytoskeleton, nuclear transport).

**Fig 1:**
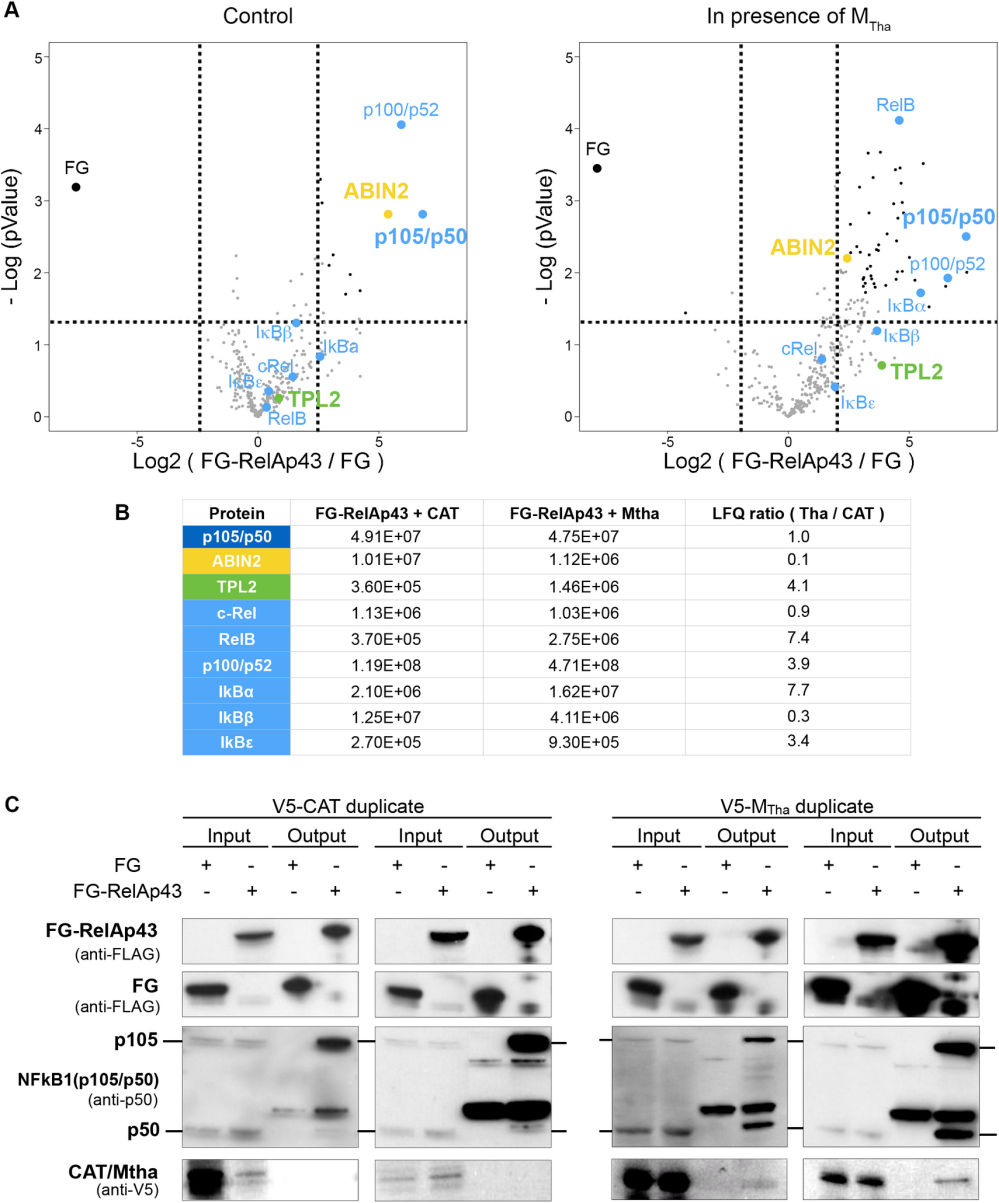
Mapping of RelAp43 interactors by MS and identification of the p105-ABIN2-TPL2 complex as a major target of M_Tha_. A. Volcano plot of FG-RelAp43 vs FG with V5-CAT (left panel) and FG-RelAp43 vs FG with V5-M_Tha_ (right panel). HeLa cells were co-transfected with 2 plasmids expressing FG or FG-RelAp43 and V5-CAT or V5-M_Tha_. After 24h, 30 μg of protein were purified by TAP and analyzed by MS. The Log2(FG-RelAp43/FG) in X-axis was obtained by a two-sample test, comparing the LFQ between FG-RelAp43 and the control FG. The Log(pValue) in Y-axis was obtained by a multi-sample test, determining if any of the means of several groups are different from each other. Each spot represent a protein significantly identified with RelAp43 (black spot) or failing the statistical analysis (grey spot). The doted lines represent the thresholds for a FDR determined at 5%. Colored spots highlight the proteins of interest (see B). B. LFQ values of the proteins of interest highlighted in (A) and ratio of LFQ values between FG-RelAp43/CAT and FG-RelAp43/M_Tha_. C. The presence of the transfected FLAG- and V5-tagged proteins as well as the endogenous p105/p50 proteins in the input and output were revealed by western blot. The results presented are 2 experiments representative of 4 replicates. The “IgH” band corresponds to the immunoglobulin heavy chain from the antibodies used in the TAP experiment.

As expected from previous work on RelAp43 [2], in absence of M_Tha_ our approach led us to identify all known NF-κB proteins except RelA, as well as IκBα and the two other major members of the IκB proteins: IκBß and IκBε (in blue in Fig 1A left). The lack of RelA identification can be explained (1) by the common RHD of RelA and its overexpressed variant RelAp43 which are undistinguishable and (2) by the presence of only 2 Lys and 1 Arg in the 207 residues constituting the specific C-ter sequence of RelA, which strongly impairs its capacity to be detected by mass spectrometry (MS) conversely to the specific C-ter part of RelAp43 (S3 Fig). Nevertheless, p100/p52, and p105/p50 were quantified as some of the most important partners of RelAp43 (Fig 1A left panel, 1B). In addition to the 49 proteins significantly interacting with RelAp43, many other proteins including 5 NF-κB / IκB proteins were identified but failed the statistical test (Fig 1A), most likely due to the low intensity of detected peptides, and could nonetheless be confidently considered in further investigation (S2 Fig). While the interaction of cRel and p105/p50 with RelAp43 is not modulated by M_Tha_, IκBß interaction with RelAp43 is reduced by a factor of 3 in the presence of M_Tha_ (Fig 1A right panel, 1B). Furthermore, IκBε, p100/p52, RelB and IκBα exhibited an increased interaction with RelAp43 by a factor of 3 to 7 in the presence of M_Tha_, RelB and IκBα becoming significantly more present. Hence, we can observe that M_Tha_ induced a strong reorganization of the interaction profiles of RelAp43 with the NF-κB and IκB proteins. Indeed, M_Tha_ led to an increased interaction between RelAp43 and RelB and p100/p52, part of the non-canonical pathway and as well as a change in its association with the regulatory proteins IκBα, IκBß and p100.

Moreover, we identified 2 proteins that are known to form a regulatory complex with p105: ABIN2 and TPL2 (S2 Fig), forming an intersection step between the NF-κB and the MAPK pathways [3]. The volcano plot (Fig 1A left panel) shows that aside from p105/p50 and p100/p52, ABIN2 was one of the most significant proteins interacting with RelAp43 in the absence of M_Tha_. However, ABIN2-RelAp43 interaction is reduced tenfold in the presence of M_Tha_ (falling under the cutoff of 5% FDR) conversely to p105/p50 which interaction remained similar (Fig 1B, S2). It is worth to note that the interaction of TPL2 with RelAp43, was found to be increased by a factor of 4 in the presence of M_Tha_ (Fig 1 right panel, 1B). Therefore, we decided to focus our work on the regulatory role of ABIN2 in a RNAT complex, and on deciphering how the activity of this complex could be modulated by the M protein of Tha virus.

### M_Tha_ favors the formation of RelAp43-p50 dimers

As TPL2 is known to regulate the processing of p105 and activation of NF-κB [5], and as ABIN2 inhibits NF-κB pathway and stabilizes TPL2, we looked in the presence of M_Tha_ for an evidence of the modification of RelAp43 interactions with p105 and p50.

To this aim, we used the samples purified by TAP and analyzed by MS. In the whole cell extract controls prior to the TAP (input), the quantity of p50 revealed by western blot is equal or slightly higher than that of p105 (Fig 1C) regardless of the presence of RelAp43 and M_Tha_. In the absence of M_Tha_ (output), the quantity of p50 is inferior to the quantity of p105 when p105/p50 is purified alongside with RelAp43. Interestingly, the quantity of p50 co-purified with RelAp43 is significantly higher in the presence of M_Tha_, similar to the quantity of p105.

Altogether these results showed a role of M_Tha_ in the modulation of the composition of a RNAT complex, resulting in the formation of RelAp43-p50 dimers.

### Characterization of the interactions between RelAp43, p105, ABIN2 and TPL2

To further confirm the existence of a RNAT complex, we used protein-protein interaction (PPI) assays such as bioluminescence resonance energy transfer (BRET) and protein-fragment complementation assay (PCA).

**Fig 2:**
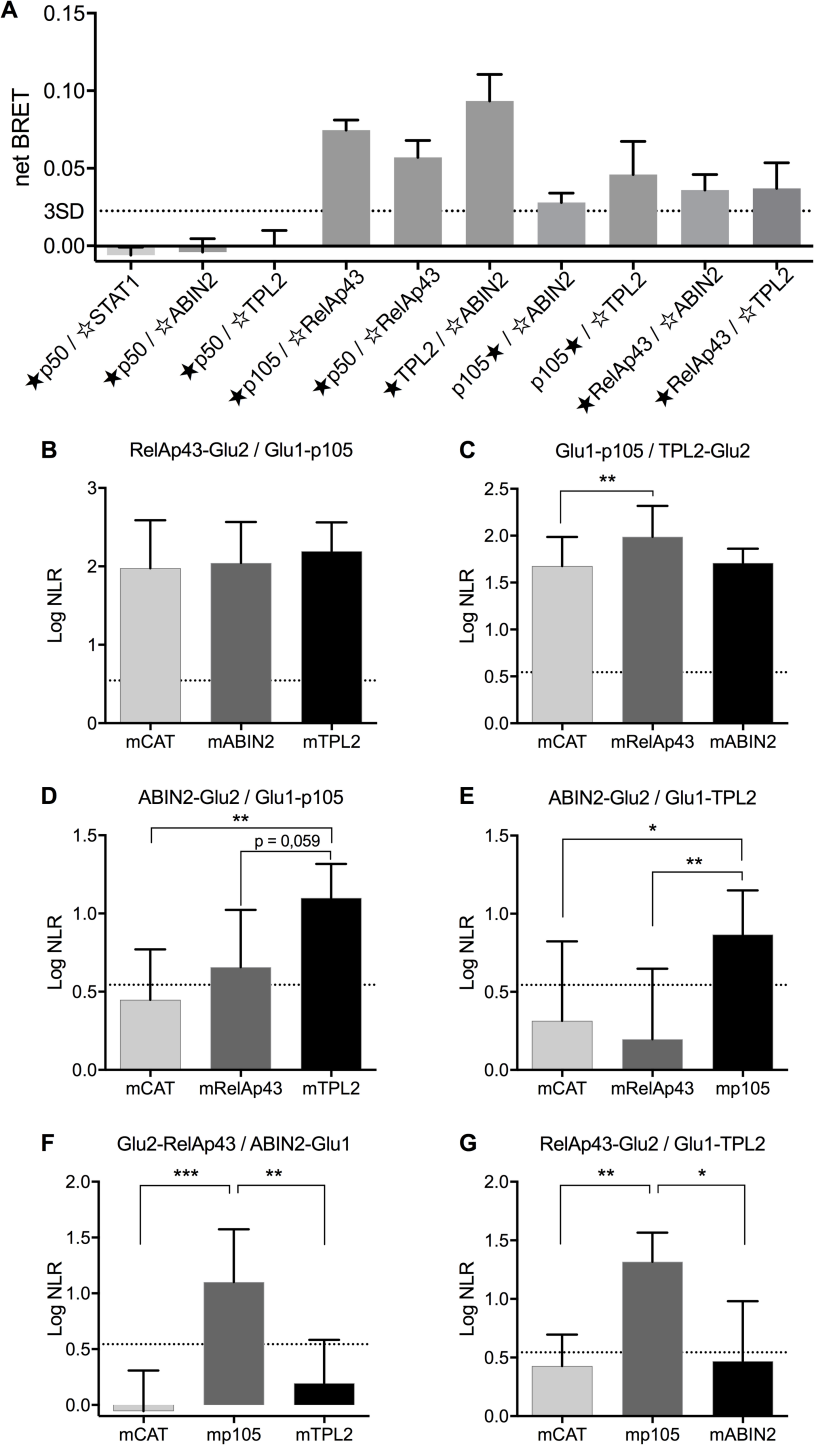
Characterization of the interactions between RelAp43, p105, ABIN2 and TPL2. A. Interactions between RelAp43, p105, ABIN2 and TPL2 assessed using BRET technology. HEK 293T cells were transfected for 48h with 2 plasmids encoding for Nluc(★)- and YFP(☆)-tagged proteins before measuring BRET. p50 and STAT1 were used as controls. A threshold (doted line) was determined at 0.025 of netBRET, based on the mean+3SD values obtained from non-interacting control pairs: p50-STAT1, -ABIN2 and -TPL2. Each value represents the mean of 3 independent experiments. Error bars represent the standard deviation. B-G. Interactions between RelAp43, p105, ABIN2 or TPL2 in presence or not of a third helper protein, using PCA technology. HEK 293T cells were transfected with 4 plasmids expressing: a Glu1-, a Glu2-, a cMyc-tagged protein and a firefly luciferase for 48h before measuring both gaussia and firefly activity. Position of the tag is indicated as follow: Glu1-, Glu2- for N-ter and -Glu1, -Glu2 for C-ter. Results are the mean of the logarithm of gaussia NLR values normalized to luciferase activity in at least 4 distinct experiments. A threshold of NLR = 3.5 previously described [54] was used to define the positive results (log 3.5 = 0.54). Error bars represent the standard deviation. ***p<0.001, **p<0.01; *p<0.05.

BRET enables identification in living cells of any interaction occurring below a 10 nm radius. STAT1, a protein from the JAK-STAT pathway which is not interacting with p50, was used as a negative control (Fig 2A). As it interacts with RelAp43 but not with ABIN2 nor TPL2 (Fig 2A), p50 was also used as a control. All negative controls were used to determine a threshold based on a three standard deviations of the mean (3SD). Hence, although the BRET signal of each interaction appeared to vary, with stronger signal for p105-RelAp43, p50-RelAp43 and TPL2-ABIN2 (netBRET>0.05) than p105-ABIN2, p105-TPL2, RelAp43-TPL2 and RelAp43-ABIN2 (netBRET<0.05), overall all combinations of interaction within the complex RNAT showed a positive result (Fig 2A).

We next study the interaction between the 4 RNAT partners using PCA (Fig 2B–2G). In comparison to BRET, here only RelAp43-p105 and p105-TPL2 interactions (stable logNLR above the 0.54 threshold) were significant (Fig 2B and 2C). This result can be explained by the high stringency required for the molecular complementation during PCA compared to the more flexible resonance energy transfer performed at nanometric distances with BRET.

In order to study more precisely the hierarchical interactions within the RNAT complex, we used PCA in the context of another third overexpressed protein (Fig 2B–2G). In these conditions, we found that the RelAp43-p105 interaction was not modified by ABIN2 nor TPL2 (Fig 2B). Similarly, a high level of interaction was observed with p105-TPL2 (Fig 2C), but in this case, it was significantly enhanced by RelAp43 (logNLR of 1.99 instead of 1.67 with the control CAT). This is more in favor of a modification of the p105-TPL2 complex induced by RelAp43 which allows the generation of a higher luciferase signal. Again, ABIN2 had not effect on p105-TPL2 complex. Moreover, a stable interaction between ABIN2 and p105 or TPL2, separately, could not be observed (Fig 2D and 2E). However, ABIN2-p105 interaction (Fig 2D) was stabilised by RelAp43 (logNLR = 0.65) and even more by TPL2 (logNLR = 1,1). In the case of the ABIN2-TPL2 interaction (Fig 2E), it was only stabilized by p105 (logNLR = 0.86). Regarding RelAp43, the collaboration of p105 was mandatory to stabilize its interaction with ABIN2 (Fig 2F) or TPL2 (Fig 2G) with a logNLR of 1.1 and 1.3, respectively. This further establishes the crucial role of p105, TPL2 and a second NF-κB protein such as RelAp43, in the initiation of the RNAT complex. Altogether, the BRET results (Fig 2A) showing the strongest signal for ABIN2-TPL2 interaction (netBRET = 0.093) and the PCA results (Fig 2B–2G), demonstrate the participation of ABIN2 to the RNAT complex through its interaction with both TPL2 and p105, p105 binding itself to RelAp43, to form a quaternary RNAT complex.

### Tha virus excludes ABIN2 from the RNAT complex

**Fig 3:**
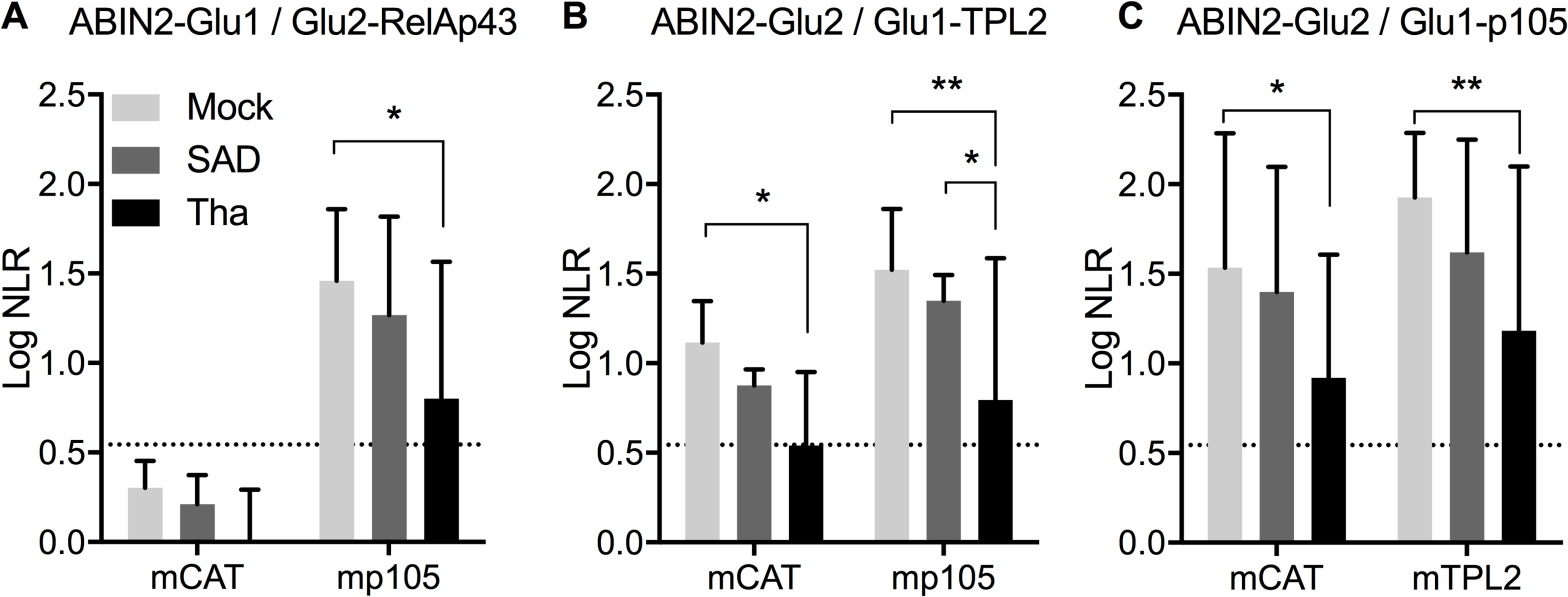
ABIN2 is excluded from a RelAp43-p105-TPL2 complex during Tha infection. Interactions between ABIN2 and RelAp43 (A), TPL2 (B) or p105 (C), using PCA technology, in combination with a third protein: mCAT, mp105 or mTPL2, and infected or not by Tha or SAD virus. HEK293T cells were infected at a MOI of 1 and transfected 3h later with 4 plasmids expressing: a Glu1-, a Glu2-, a cMyc-tagged protein and a firefly luciferase for 48h before measuring both gaussia and firefly activity. Position of the tag is indicated as follow: Glu1-, Glu2- for N-ter and -Glu1, -Glu2 for C-ter. Results are the mean of the logarithm of gaussia NLR values normalized to luciferase activity of at least 4 distinct experiments. A threshold of NLR = 3.5 previously described [54] was used to define the positive results (log 3.5 = 0.54). Error bars represent the standard deviation. **p<0.01; *p<0.05.

In order to confirm the role of rabies virus proteins in the modulation of the composition and activities of the RNAT complex and more specifically on interactions involving ABIN2, we performed a PCA using 3 partners, as described in Fig 2B, but in the context of viral infection (Fig 3). To do so, we used the Tha and SAD viruses which M proteins were previously described as differentially targeting the NF-κB pathway [2].

In these conditions, we confirmed that the interaction between ABIN2 and RelAp43 is facilitated by p105 (Fig 3A). However, the infection with Tha virus significantly (p<0.05) decreased this interaction even in the presence of p105, when compared to the mock infected cells (logNLR dropped from 1.45 to 0.80), confirming the results from MS. Regarding ABIN2 and TPL2 interaction (Fig 3B), Tha virus infection reduced the interaction of the two proteins in absence of p105 compared to the control cells (logNLR dropped from 1.11 to the level of the threshold set at 0.54). This effect is even more striking in cells overexpressing p105, where a strong and significant decrease of the interaction between ABIN2 and TPL2 is noticed (from 1.52 and 1.34 in the mock, p<0.01, and SAD, p<0.05, infected cells to 0.80 logNLR with Tha, Fig 3B). In the case of ABIN2 and p105, the interaction was decreased in Tha compared to mock infected cells, in absence (1.53 to 0.91 logNLR) as well as in presence (1.92 to 1.18 logNLR) of overexpression of TPL2 (Fig 3C). Overall, this indicates that Tha virus can strongly disturb the interaction of ABIN2 with the other members of the RNAT complex.

### M_Tha_ interacts with the RNAT complex

**Fig 4:**
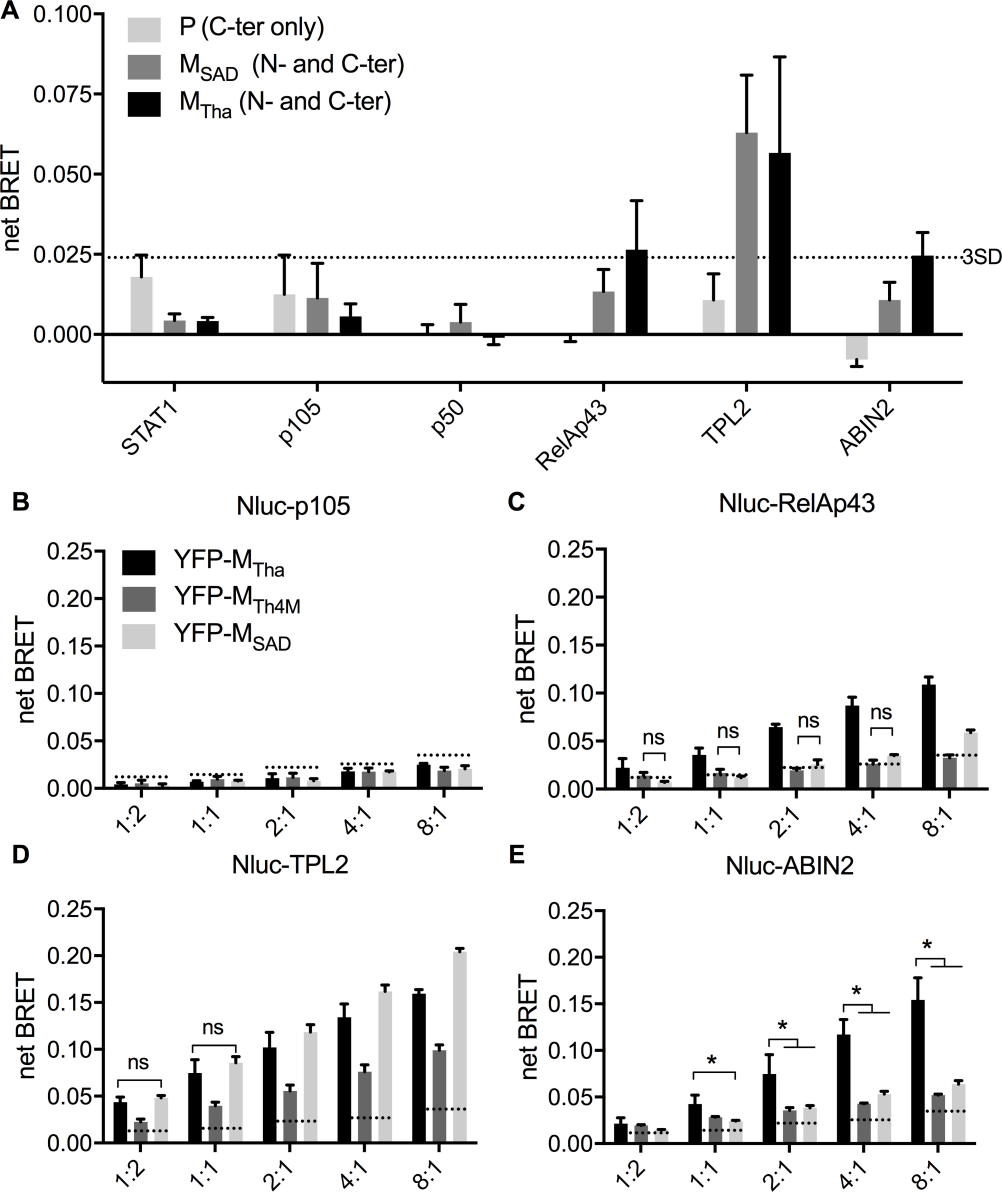
M_Tha_ interacts with RelAp43 and ABIN2. A. Interactions between M_Tha_, M_SAD_ or P and RelAp43, p105, p50, ABIN2 and TPL2 were assessed using BRET technology. P and STAT1 were used as controls. BRET was measured in living HEK 293T cells 48h after transfection. Each value is the mean of all the combinations between Nluc-tagged host proteins and YFP-tagged viral proteins in N- and C-ter position of each protein (excepted P that was only tagged in C-ter), and performed in duplicate. A threshold (doted line) was determined at 0.024% of BRET, based on the mean+3SD values of the M protein-STAT1 and P protein-RelAp43, -p105, -p50, -ABIN2 and -TPL2 pairs of partners. Error bars represent the standard deviation. B-E. M_Tha_, M_Th4M_ and M_SAD_ interacting with p105 (B), p43 (C), TPL2 (D), and ABIN2 (E), using BRET technology. BRET signal was measured in living HEK 293T cells 48h after transfection of plasmids expressing YFP- and Nluc-tagged proteins at 5 ratios of DNA, from 1:2 to 8:1. A threshold (doted line) was determined for each dilution of DNA (1.2, 1.4, 2.2, 2.5, 3.4% of BRET respectively), based on the mean+3SD values of the M proteins-p105 pairs of partners. Each value is the mean between 3 experiments with error bars representing the standard deviation. *p<0.05. For D, only the non significant (ns) values were highlighted.

Next, we investigated the capacity of the M protein of Tha and SAD to interact with the RNAT complex using the BRET technology. Both STAT1, which doesn’t interact with the M proteins, and the P protein, which doesn’t interact with the proteins RelAp43, p105/p50, ABIN2 or TPL2, were used as negative controls to determine a 3SD threshold. Since the P protein can interact with phosphorylated STAT1 [20], this pair was not considered as a negative control. None of the M proteins lead to a significant interaction (higher than 3SD) with STAT1, p105 and p50 (Fig 4A). However, M_Tha_ but not M_SAD_ seems to interact with RelAp43, which is expected [2], but also with ABIN2. Both, M_Tha_ and M_SAD_ gave a strong positive signal with TPL2 (netBRET > 0.05).

To further investigate the interactions between the M proteins and each of the members of the RNAT complex, we included a M_Tha_ protein mutated on the positions 77, 100, 104, 110 (M_Th4M_) and showing a loss of interaction with RelAp43 (Fig 4C) [19]. Based on the absence of significant interaction with M proteins, p105 (Fig 4B), p50 and STAT1 (S4A Fig) were used as negative controls. They did not show any significant BRET activity, even while using higher YFP:Nluc ratios (S4B Fig), which are known to increase the potential efficiency of BRET [21,22]. At the opposite, the M proteins exhibited a strong increase of the BRET signal significantly above the controls with RelAp43, TPL2 and ABIN2 (Fig 4C–4E). Fig 4C confirmed the ability of interaction of M_Tha_ with RelAp43 (netBRET ranging from 0.022 to 0.109) while both M_Th4M_ and M_SAD_ presented a very low netBRET if any (ranging from 0.001 and 0.006 to 0.003 and 0.059, respectively). Moreover, the observation of a significant difference at the 1:2 ratio, where the efficiency of BRET is low, is a strong evidence of this interaction. As expected, all M proteins interacted with TPL2 (Fig 4D), although M_Th4M_ showed for unknown reason a netBRET about half that of M_Tha_ and M_SAD_. More interestingly, Fig 4E highlights that M_Tha_ interaction with ABIN2 (increasing from 0.042 to 0.154 of netBRET) is strongly higher than that of M_Th4M_ or M_SAD_ (increasing from 0.028 and 0.022 to 0.052 and 0.063 of netBRET, respectively). Overall, this shows that M protein interaction with the NF-κB pathway is more complex than previously described [2,19]. While we highlight that M proteins can also interact with TPL2, a distinct interaction of the M protein from a field isolate virus with ABIN2—similarly to RelAp43—is shown for the first time.

### Tha virus modulates the inflammation

**Fig 5:**
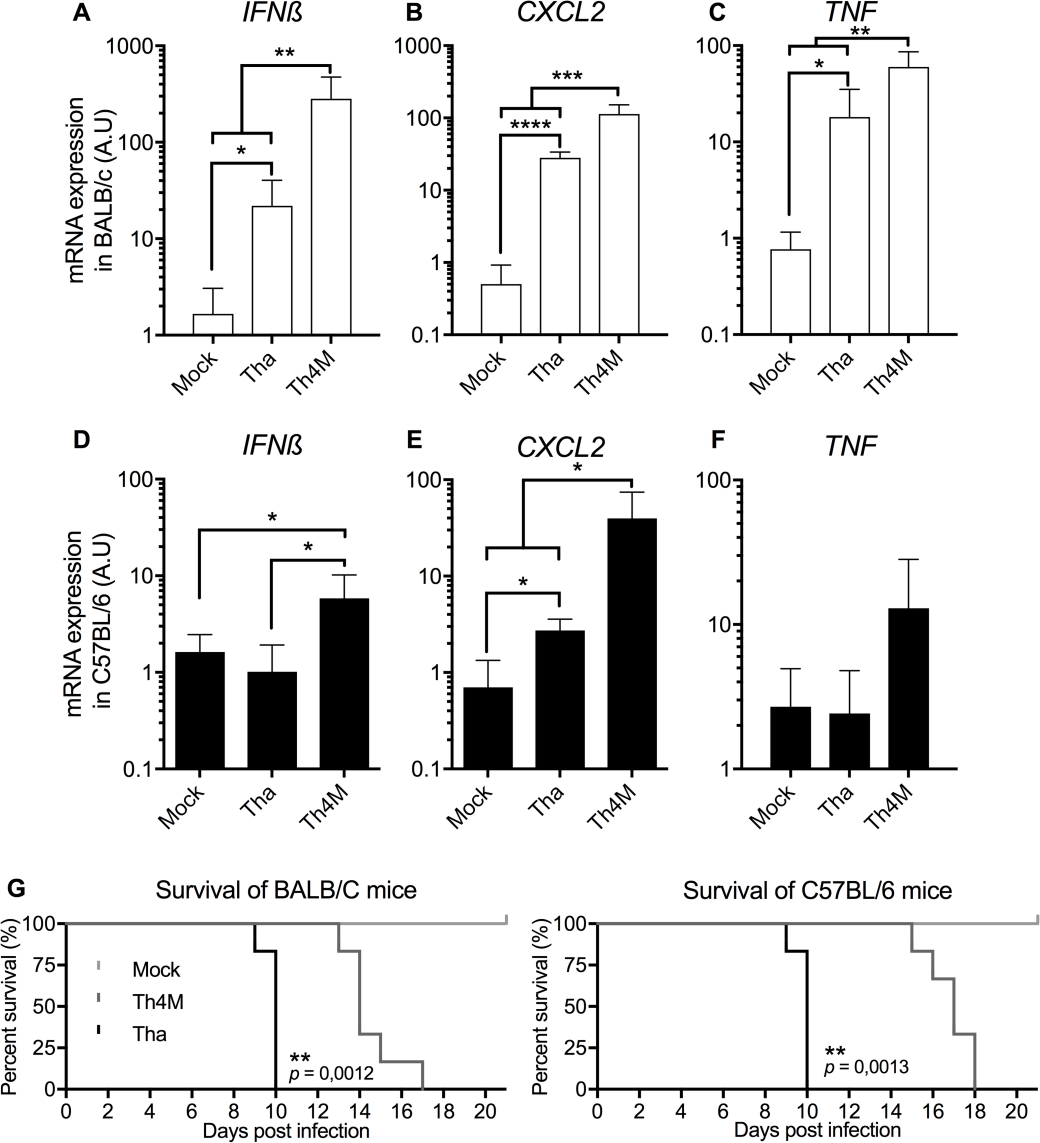
Tha virus modulates the response to the infection in infected mice. A-F. Relative quantification of *IFNß* (AD), *CXCL2* (BE), and *TNF* (CF) mRNA expression in the brain of mice infected by Tha or Th4M viruses. Six weeks old BALB/c (CDE) or C57BL/6 (FGH) were infected by intramuscular injection and monitored over 21 days. The mice were sacrificed at experimental end point and mRNA was extracted from the brain. Six mice were used per condition. Error bars represent the standard deviation. G. Survival curve of the BALB/c (left panel) and C57BL/6 (right panel) mice used for RNA quantification in A-F.

NF-κB signaling is involved in the induction of the immune response and its control by the M protein is already well established. While M_SAD_ and M_Th4M_ can induce NF-κB activity, M_Tha_ is able to strongly inhibit its activation [2,19]. Hence, we quantified the expression of various immunity-related genes regulated by NF-κB: *IFNß*, *CXCL2*, *TNF* (Fig 5A–5F*)*, *CCL5* and *CXCL10* (S5A–S5D Fig) in the brain of mice from two genetic backgrounds [23,24], at late stage of the infection by Tha or the isogenic Th4M virus mutated on the positions 77, 100, 104, 110 of the M protein [19]. Tha and Th4M virus have similar replication rates in the brain of mice, both at 9 days after infection and at the experimental end point (S6B Fig). *IFNß*, *CXCL2* and *TNF* have already been shown to be modulated by the interaction of M_Tha_ with RelAp43 *in cellulo* [19].

After 9 days of infection, neither Tha nor Th4M induce the expression of *IFNß*, *CXCL2* or *TNF* in BALB/c (S6C–S6E Fig). However, at the experimental end point, both Th4M and Tha virus induce an increase of the expression of *IFNß*, *CXCL2* and *TNF* in BALB/c mice (Fig 5A–5C) while almost no induction is observed in C57BL/6 mice infected by Tha virus compared to Th4M (Fig 5D–5F). Indeed, Th4M virus induced a stronger increase of the expression of *IFNß* (Fig 5A and 5D), *CXCL2* (Fig 5B and 5E) and *TNF* (Fig 5C and 5F) compared with Tha (13-, 4- and 3.3-folds in BALB/c, respectively; 5.8-, 14- and 5.3-folds in C57BL/6 mice, respectively). In comparison, both Tha and Th4M viruses induce a strong expression of *CCL5* and *CXCL10* (S5 Fig) and no differences between the two viruses could be observed. Therefore, if CCL5 is overexpressed under SAD infection compared to Tha infected cells [19], this pattern seems to be due to a higher stimulation of the immune response than an inhibition by M_Tha_ and its control together with that of CXCL10 must be strictly restricted to the N protein of pathogenic Ni virus [25].

Interestingly, late infection symptoms (corresponding to experimental end point) appeared in Tha infected mice within 10 days post-infection (dpi) regardless of the genetic background (Fig 5G), while appearance of the late infection symptoms in Th4M infected mice was delayed by 4 to 8 days (median of survival at 14 and 17 dpi in BALB/c and C57BL/6, p<0.05, respectively).

Overall and although some differences exist between the two genetic background [23,24], those results suggest that Tha virus could exert a repression of the expression of inflammatory related genes such as *IFNß*, *CXCL2* and *TNF* in the brain of infected mice at the late stage of the infection compared with Th4M while not affecting CCL5 and CXCL10. Moreover, the mutation on the positions 77, 100, 104, 110 of the M protein of Tha virus, which leads to a stronger immune response to the infection, can also be correlated with a higher survival rate of the infected mice.

## Discussion

### New insights on the interactions within NF-κB pathway and the p105, ABIN2 and TPL2 complex

The capacity of the NF-κB pathway to control various responses derives from the differential regulation of a wide range of target genes through the formation of a broad NF-κB dimer repertoire [26]. A splicing variant of RelA, RelAp43, was recently involved in the control of the immune response during rabies virus (RABV) infection [2,19] and forms complexes with all the NF-κB and IκB proteins. Amongst them, the regulatory proteins p105/p50 and p100/p52 are the most significant NF-κB proteins interacting with RelAp43. While active NF-κB dimers are mainly composed of RelA-p50 dimers, RelA is also involved in the regulation of p100/p52. The regulatory p100 precursor serves as an inhibitor of p65/RelA as RelA-p100 dimers are not active after TNF stimulation [27] and RelA-p52 dimers have been shown to be part of several signaling pathways [28]. Therefore, RelAp43 could act as an important competitor of RelA in both canonical and non-canonical pathways, according to the physiological context.

**Fig 6:**
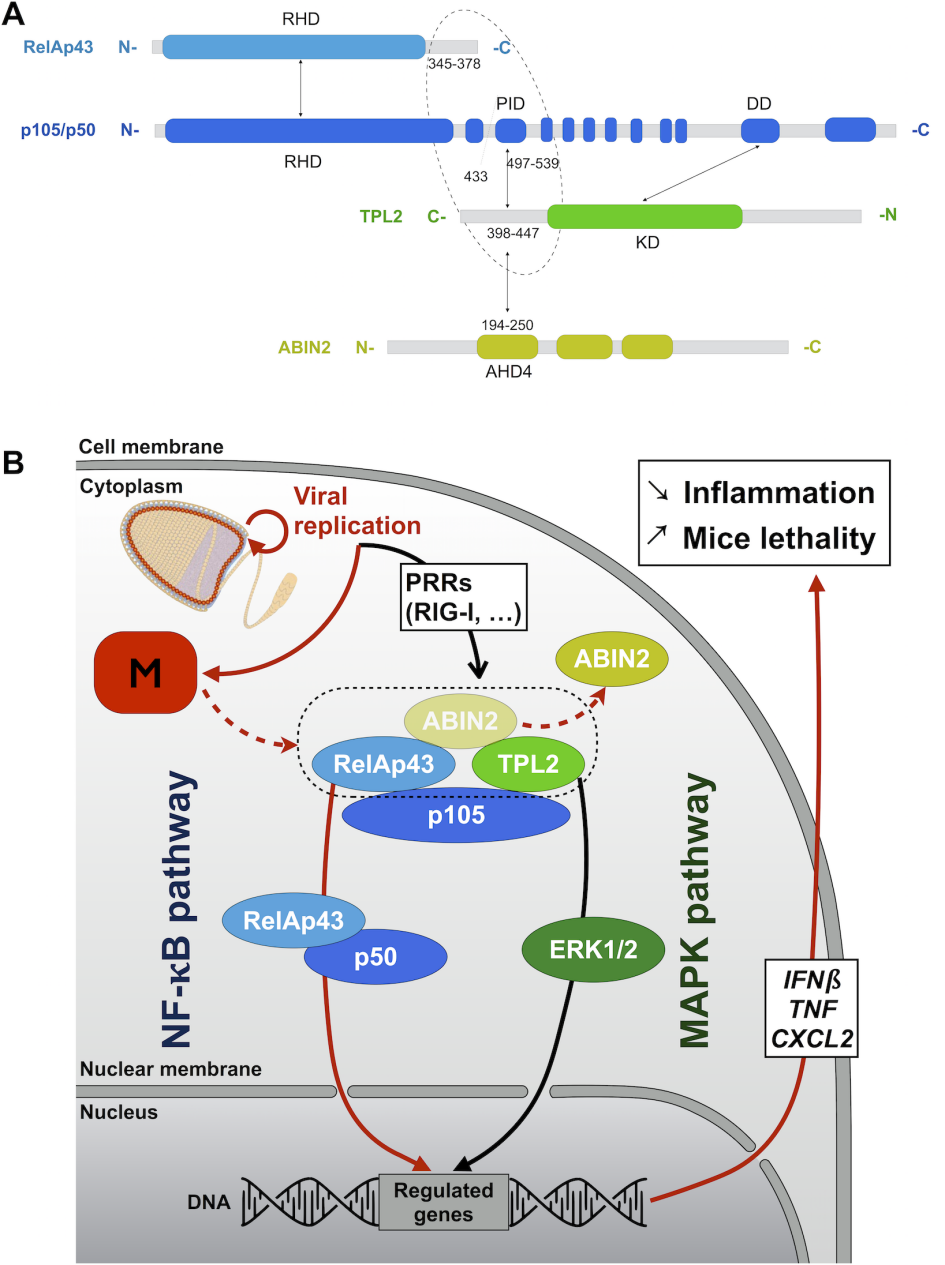
Model of the control of the RelAp43-p105-ABIN2-TPL2 complex by Tha virus to fine-tune the response to the infection. A. Interactions between RelAp43, p105, ABIN2 and TPL2 adapted from [3]. RelAp43 and p105 interact through their respective RHD domain. p105 interact with its C-ter part with TPL2 on two different domains: the processing inhibitory domain (PID) of p105 with the C-ter part of TPL2 and the death domain (DD) of p105 with the kinase domain (KD) of TPL2. ABIN2 interacts with TPL2 through its ABIN-homology domain 4 (AHD4) with the same C-ter region of TPL2 involved in the interaction between p105 and TPL2. The circle highlights a region where each protein of the complex might be in a very close proximity, possibly involved in the binding of ABIN2. B. The RelAp43-p105-ABIN2-TPL2 complex is modulated by the matrix protein of pathogenic rabies virus. M proteins destabilize the interaction of ABIN2 with the complex which leads to the regulation of both the NF-κB and the MAPK dependent genes in response to viral infection.

In addition, all the partners of the ternary complex p105-ABIN2-TPL2, regulating the activation of downstream NF-κB and MAPK pathways [3] are associated to RelAp43 within a close range (< 10 nm). The mapping of PPIs showed TPL2 to be mainly responsible for the interaction of ABIN2 with the complex while co-immunoprecipitation experiments showed that ABIN2 preferentially forms a larger ternary complex including TPL2 and also p105 [3]. Interestingly, the C-ter region of TPL2 involved in the interaction with the processing inhibitory domain (PID) of p105 (which also mediates p105 dimerization) is also involved in the interaction with the 194–250 region of ABIN2 (Fig 6A) [3]. Hence, both ABIN2 and p105 interact with one singular domain of TPL2. Investigating close proximity interactions, we shed a new light on the specific role of the different proteins in the complex (Fig 6A). ABIN2 is the member of the complex exhibiting the weaker interaction with the others. Overall, this suggests that p105 and TPL2 might form together a region including the C-ter of TPL2 and the PID of p105 securing a stable interaction with ABIN2.

Finally, RelAp43 interacts with p105 most likely through the RHD [29] and helps its association with TPL2 and therefore of ABIN2 with the complex. All observed and reported interactions are summarized in S1 Table and Fig 6A. Interestingly, RelAp43 and ABIN2 are also able to interact with the help of p105 but without the overexpression of TPL2. This suggests two possibilities. RelAp43 could allow this interaction without TPL2 as it exhibited a small signal of interaction between ABIN2 and p105. Another explanation could be that the endogenous TPL2 is sufficient to bring ABIN2 to the RNAT complex. Altogether, this suggests that RelAp43 affects p105 and stabilize the formation of the complex. It is likely that while p105 is mandatory to bring ABIN2 to NF-κB dimers, several members of the NF-κB family can form a complex with p105-ABIN2-TPL2 [5,30]. Whether the capacity of p105 to form a complex with TPL2 and ABIN2 increases solely when dimerized with RelAp43 and its specific C-ter part or whether any other NF-κB proteins can contribute remains to be determined. However, crystallization of RelA-p50 dimers [29] suggests that the RelAp43 C-ter region should be nearby the PID region of p105 and therefore, close to the suggested pocket of interaction between p105, ABIN2 and TPL2 (Fig 6A).

Here we confirmed that ABIN2 forms a stable interaction within close proximity only with both p105 and TPL2. Moreover, while it has been suggested so far that the p105-ABIN2-TPL2 complex is separated from the main NF-κB pathway and uses a separate pool of p105 [3], here we show that the assembly of this ternary complex can be favored by a second NF-κB protein such as RelAp43 and that all proteins interact below 10 nm of distance of each other as shown by BRET.

### M_Tha_ disturb the NF-κB network of RelAp43 and the RNAT complex

The central role in cell homeostasis of the NF-κB pathway makes it a target of choice for viruses such as RABV to control the immune response and help it to silently spread within the host [10]. In the case of pathogenic lyssaviruses, such as the Thailand strain, the M protein interacts with RelAp43 and disturbs the homeostasis within the NF-κB dimers. M_Tha_ could lock RelAp43-dependent NF-κB dimers with IκBα in an inactive state, as well as favor its interaction with the non-canonical pathway. In the case of RelB, it has been well established that the formation of RelA-RelB dimers inhibit both RelA and RelB respective pathways by inhibition of the DNA binding [31–33]. Hence, it could be interesting to investigate the DNA binding capacity of a RelAp43-RelB dimer, as the enhancement of the interaction between RelAp43 and RelB by M_Tha_ could lead to a differential regulation of the canonical and non-canonical pathway.

Although firstly described as having a most likely direct interaction with RelAp43, here we show the capacity of M_Tha_, but also in a lower extent of M_SAD_, to interact with a cluster of NF-κB signaling proteins (S1 Table). Indeed, if the capacity of the M protein to interact with TPL2 is conserved in both attenuated and pathogenic viruses, only the M protein of a pathogenic virus seem able to significantly interact with both RelAp43 and ABIN2. The M protein is then likely to first dock on TPL2, and interact afterward with ABIN2 and/or RelAp43, two properties that are lost in attenuated viruses. Therefore, this questions the effect of the single interaction of M with TPL2 during the infection by attenuated virus on cell signaling. However, as the capacity of M_Tha_ to disturb the NF-κB pathway was shown to depend on RelAp43 expression [19], the interaction of M_Tha_ with only ABIN2 and TPL2 is not enough to disturb it.

M_Tha_ significantly enhances the interaction of many proteins with RelAp43 complexes (Figs 1 and S2) and interacts with 3 out of the 4 proteins of a single complex: RelAp43, ABIN2 and TPL2. Even if the M protein is known for having pleiotropic properties, the specific interaction of M_Tha_ with such a wide range of host proteins is highly unlikely. As the M protein of lyssaviruses is known to oligomerize [34] and form super structures within the cell [15], we suggest that M_Tha_ can act as a scaffolding agent for the formation of high molecular weight complexes, targeting several cellular function at the same time (S2 Fig). Additionally, a region of the M protein (positions 33–36) was described as sharing the features of a proline-rich motif (PRM) and interacts with the position 107–112 on a second M protein and forming a hydrophobic cleft [34]. PRMs are common motifs amongst host protein-protein interactions and their hydrophobic properties could facilitate the binding to many host proteins.

At this stage we observed that (1) the MS quantification of ABIN2 purified with RelAp43 is reduced by 10 fold in presence of M_Tha_, which goes against the general trend of M_Tha_ enhancing interactions, (2) the M_Tha_-ABIN2 interaction is strikingly stronger than that of M_Th4M_ or M_SAD_ and, (3) the Tha virus has a higher propensity to weaken the interaction of ABIN2 with RelAp43, TPL2 and p105 compared to the SAD virus and mock infected cells. This leads strongly to the hypothesis that M_Tha_ disturb the RNAT complex, weakening the capacity of interaction between ABIN2 and the hypothesized region of interaction formed by p105, TPL2 and maybe RelAp43 (Fig 6A). Interestingly, our results demonstrate a switch of the interaction of the M protein between a pathogenic and a vaccinal strain.

Further investigation should also focus on ABIN2 and its role as an inhibitor of RIP1, upstream of NEMO (which were not identified by MS) and the NF-κB pathway [35]. A working hypothesis is that ABIN2 could be recruited to polyubiquitin chains when it is released from activated TPL2, restricting the activation of innate immune signaling networks [36]. Therefore, as we have shown a strong interaction of M_Tha_ to ABIN2, M_Tha_ could interfere with the NF-κB signaling at several levels through its interaction with ABIN2. Furthermore, ABIN2 has been linked to the ESCRT pathway [37], and its interaction with the M protein could be related to RABV budding [38].

### Tha virus controls cell signaling to fine-tune the host response to the infection

While destabilizing the RNAT complex leading to the exclusion ABIN2, M_Tha_ controls downstream NF-κB and MAPK pathways. Indeed, M_Tha_ facilitates the formation of RelAp43-p50 NF-κB dimers lacking a TAD which can regulate the expression of NF-κB targets [19]. To do so, M_Tha_ either induce directly the processing of p105 into p50 in a RelAp43 dependent manner, or modify the homeostasis of NF-κB dimers [5]. This remains to be clarified. It is worth to mention that the production and/or release of p50 from the cytoplasm and translocation of an active NF-κB dimer (not including RelA) was observed under infection by the laboratory strain CVS [39]. Together with our results, it shows that p50 could have an important role in rabies virus infection that should be further investigated.

In parallel, MS results suggest that TPL2 is being stabilized without ABIN2, implying that M_Tha_ could lock TPL2 on p105 (Fig 6, S1 Table). Yet, the implications on TPL2 remain to be investigated using time sensitive approaches to determine if and in which order M_Tha_ destabilizes, liberates or blocks TPL2 as well as the effects on downstream activity on MAPK ERK1/2 and MAPK-dependent transcription factors [3].

Hence, in addition to P protein control of IRF and JAK-STAT pathways during RABV infection [10], M appears to have a central function in cell signaling inhibition and modulation of innate immune response through the control of TPL2, a key regulator of NF-κB and MAPK pathways. It is worth to note that TPL2 regulates also IRF proteins, leading to a strong induction of IFNβ during vesicular stomatitis virus (VSV) infection [40] and appears downstream of JAK-STAT pathway, holding an essential role in antiviral host defense against influenza virus infection [41]. Therefore, the perturbation of TPL2 signaling by M could be potentially implicated in a much further control of the host response, which could be conserved across pathogenic and attenuated viruses.

The NF-κB pathway is involved in the expression of several genes of the immune and inflammation response to the infection such as *IFN*β, *CXCL2* and *TNF* [8,42]. The modulation of their expression by Tha virus depending on the capacity of the M protein to interact with RelAp43 [19], as well as ABIN2, is here confirmed in Tha infected mice. Interestingly, IFNβ, CXCL2 and TNF expression control by the M protein is observed in the late stages of the infection *in vivo*, further corroborating the modulation by M_Tha_ of the immune response observed in cellulo [19]. Further, it would be interesting to explore the effect of M_Tha_ hijacking of the NF-κB signaling on other NF-κB-dependent genes, possibly involved in RABV pathogenesis. Additionally, the control of the expression of such genes mainly by the NF-κB signaling pathway, but also potentially in synergy with other pathways, correlates with the incubation and pathogenicity of RABV. Hence, through the regulation of the NF-κB pathway by the M protein, Tha virus is able to fine-tune the host response to the infection.

### Conclusion

RelAp43 and the matrix protein of pathogenic rabies virus are known to interact and modulate the innate immune response [2,19]. However, we show here that the mechanisms involved are far more complex than initially thought. M_Tha_, and to a lower extent M_SAD_, can interact with RelAp43 and other proteins involved in NF-κB as well as MAPK signaling. While introducing a new insight in the NF-κB network and especially in a p105-regulating complex, we gathered for the first time evidences suggesting that a pathogen, rabies virus, can use one of its proteins (the M protein) to modulate the interaction of ABIN2 with p105 and TPL2. Thereby, the M protein leads to the control the NF-κB pathway in order to modulate the inflammation in response to RABV infection.

## Material and methods

### Cells and viruses

Human carcinoma epithelial cells (HeLa, ATCC CCL2™), human epithelial kidney cells (HEK-293T/17, ATCC CRL-11268™) are part of the collection of our laboratory and were cultured as previously described [17]. Virus infection was performed in 6- or 96-well plate dishes during indicated times at 37°C and using different viruses at a multiplicity of infection (MOI) of 1. Thailand virus, referred as Tha (isolate 8743THA), is a field strain of RABV isolated in Thailand from a human bitten by a dog (EVAg collection, Ref-SKU: 014V-02106). SAD-B19 virus (SAD) is a vaccine strains of RABV (EVAg collection, Ref-SKU: 014V-02283). A recombinant Tha virus mutated on the positions 77, 100, 104, 110 of the matrix protein (Th4M) was used as previously described [19].

### Plasmids

The coding sequence (CDS) of CAT, M_Tha_, M_SAD_, M_Th4M_, RelA and RelAp43 were obtained from pcDNA3.1N-V5-dest plasmids in the laboratory [2]. The CDS of NFκB1(p105/p50) was given by R. Weill (Institut Pasteur). The CDS of TPL2 and ABIN2 were amplified by PCR from cDNA obtained from HeLa cells. The CDS were compared to the RefSeq of their respective variant number 1: NM_005204.3 and NM_024309.3 (NCBI). Genes were inserted using In-Fusion technology (Clontech) in various plasmids. pKmyc vector was a gift from Ian Macara (Addgene plasmid #19400) to add the c-Myc tag in N-ter position. Gaussia Luciferase-Based Protein Complementation Assay (PCA) plasmids to add a Glu1 or Glu2 tag in N- or C-ter position of the insert were a gift from D. Gerlier [43]. A modified version of the pEGFP-C1 plasmid (Promega) into p3xFLAG-EGFP-C1, presenting a 3xFLAG tag upstream to the eGFP[44] was a gift from F. Thierry (Institute of Medical Biology, Singapore). It adds a FG (3xFLAG-eGFP) tag in N-ter position. The pEYFP-C1/N1 plasmids (Promega) were commercially purchased, adding a eYFP tag in N- or C-ter position. The pEYFP-C1/N1 plasmids were modified by switching the eYFP to the Nano-Luciferase (Nluc) to obtain new pNluc-C1/N1 plasmids, adding a Nluc tag in N- or C-ter position. All sequences were controlled by sequencing, using Sanger technology. All constructions are summarized in S2 Table. Finally, the plasmid pGL4.50 (Promega) expressing the Firefly Luciferase was used as control.

### Tandem affinity purification and mass spectrometry

HeLa cells were plated onto 75 cm^2^ dishes with 2.10^6^ cells per dish in 15 mL of medium (10 dishes per condition). After 24h, cells were transfected with 6 μg of FG or FG-RelAp43 plasmids and 4 μg of V5-CAT or V5-M_Tha_ plasmids using Lipofectamine 2000 (Invitrogen). Cell pellets were lysed 24h later in a FLAG Buffer (150 mM Tris-HCl, 300 mM NaCl, 1% Triton-100X). 30 mg of protein extract were incubated overnight at 4°C on anti-FLAG-M2 beads (Sigma) in FLAG Buffer. Protein complexes were then eluted with 3xFLAG purified peptides (Sigma) and incubated for 1 h with GFP-Trap_A (Chromotek) in a GFP Buffer (50 mM Tris-HCl pH 7.4, 150 mM NaCl, 0.2 mM EDTA). Proteins were finally eluted directly using loading sample buffer (Invitrogen) and heated at 95°C for 10 min. After centrifugation, a quality control of the eluted proteins was performed by western blot. Each condition was done in 3 biological replicates.

dx.doi.org/10.17504/protocols.io.jeqcjdw [PROTOCOL DOI].

Protein samples were loaded on a SDS-PAGE gel (4–12% gradient, Biorad). After the electrophoretic migration the gel was stained with Coomassie Blue R-250 (Biorad) and each lane was cut into 10 gel bands. Gel slices were washed twice with 100 mM ammonium bicarbonate for 15 min, followed by 100 mM ammonium bicarbonate/acetonitrile (1:1) for 15 min. After reduction and alkylation, proteins were digested by 0.5 μg of modified sequencing grade trypsin (Promega, Madison, WI, USA) in 10 mM ammonium bicarbonate overnight at 37°C.

Resulting peptides were extracted from the gel by incubation in 50 mM ammonium bicarbonate for 15 min, and three times in 5% formic acid (FA) and 50% acetronitrile (ACN) for 15 min. All extractions were pooled and dried down in a vacuum concentrator, and further resuspended in 2% acetonitrile, 0.1% FA before injection.

Trypsin-digested peptides obtained for all gel slices were analyzed separately by nanoLC-MS/MS using an UltiMate 3000 RSLC (Dionex, Amsterdam, The Netherlands) coupled to an LTQ-Orbitrap Velos mass spectrometer (Thermo Fisher scientific, Bremen, Germany). Five μL of each sample were loaded on a C_18_ pre-column (300 μm inner diameter × 5 mm; Dionex) at 30 μL/min in 2% ACN, 0.1% FA. After 4 min of desalting, the pre-column was switched online with the 15 cm capillary column (75 μm diameter filled with 3 μm Reprosil-Pur Basic C_18_-HD resin) (Dr. Maisch GmbH, Ammerbuch-Entringen, Germany) equilibrated in 98% solvent A (2% ACN, 0.1% FA) and 2% solvent B (80% ACN, 0.08% FA). Peptides were eluted using a 2 to 55% gradient of solvent B during 30 min at 300 nL/min. The LTQ-Orbitrap Velos was operated in data-dependent acquisition mode with the XCalibur software. Survey scan MS were acquired in the Orbitrap in the 300–2000 m/z range with the resolution set to a value of 60,000 at m/z = 400. The 10 most intense ions per survey scan were selected for collision-induced dissociation (CID), and resulting fragments were analyzed in the linear trap (LTQ). Dynamic exclusion was employed within 20 s and repeated during 30 s to prevent repetitive selection of the same peptide.

### Data processing and analysis

Raw files were processed with Maxquant [45] (v.1.4.1.2) and the Human Swiss-Prot FASTA database (20,240 proteins) concatenated with 4 recombinant proteins (FG, FG-RelAp43, V5-CAT and V5-Mtha) was used. Andromeda searches [46] were performed choosing trypsin as specific enzyme with a maximum number of 2 missed cleavages. Possible modifications included carbamidomethylation (Cys, fixed), oxidation (Met, variable) and Nter acetylation (variable). The mass tolerance in MS was set to 20 ppm for the first search then 6 ppm for the main search and 0.5 Da for MS/MS. Additional peptides were identified by the “match between run” option with a maximal retention time window of 1 min. Five amino acids were required as minimum peptide length and 1 unique peptide was required for protein identification. A false discovery rate (FDR) cutoff of 1% was applied at the peptide and protein levels. MaxLFQ, Maxquant’s label-free quantification (LFQ) algorithm was used to calculate protein intensity profiles across samples [47]. A minimum peptide ratio count of 2 was required for LFQ calculation (S2A Fig).

For statistical and bioinformatics analysis, as well as for visualization, Perseus, which is part of Maxquant, was used [48].

The “proteinGroup.txt” file was processed as described in Tyanova et al. 2016. Two valid LFQ values out of three were required for a confident quantification across all replicates. Protein LFQ intensities were logarithmised and missing values imputed by values simulating noise around the detection limit. For pairwise comparison and identification of interacting proteins, t-test statistics were applied with a permutation-based FDR set to 5% and a S0 of 2 [49].

Protein-protein interaction networks for genes of interest were obtained using STRING v10 [50]. Interactions were determined with the following sources: experimentally determined, automated text mining and database annotations (minimum score: 0.9). Finally, the network was visualized with Cytoscape [51] and clusters were annotated using DAVID v6.8 [52,53].

### Western blot and antibodies

Western blot analysis was performed using NuPAGE gels (Invitrogen). Protein transfer on nitrocellulose membrane was performed using iBlot transfer system (Invitrogen), as indicated by provider. Membranes were saturated for 1 h in PBS-Tween 0.1% with 5% non-fat dried milk. Immunoblotting procedure consisted in overnight incubation with indicated primary antibody diluted in 5% dried milk PBS-Tween, washed three times for 5 min in PBS-Tween, then incubated 1 h with indicated HRP conjugated secondary antibody. The following antibodies were used: mouse a-V5 antibody (Invitrogen); mouse a-FLAG M2 antibody (Sigma), mouse a-p50 antibody (SantaCruz), HRP-linked a-mouse antibody and HRP-linked a-rabbit antibody (GE Healthcare). Blots were revealed by chemiluminescence and exposure to X-ray films or with an imager (Amersham) for different time to avoid saturation.

### Protein-fragment complementation assays (PCA)

HEK-293T cells were plated in 96-well plates with 25 000 cells per well in 100 μL of culture medium. After 24h, cells were transfected with Lipofectamine 2000 with 2 plasmids, each of them expressing either Glu1 or Glu2 (N- or C-ter tagged) recombinant protein, a plasmid expressing a cMyc-tagged protein and a control plasmid expressing the Firefly Luciferase. If specified, cells were infected 3 hours before transfection. After cell lysis, the Gaussia and Firefly activity were measured separately using respectively the Renilla Luciferase kit and the Firefly Luciferase kit (Promega). After a first normalization with the Firefly activity, Normalized Luminescence Ratio (NLR) was obtained from split Gaussia luciferase’s activity using the following formula (1), as previously described [54].

NLR=signal(Glu1-A+Glu2-B)/[signal(Glu1-A+Glu2)+signal(Glu1+Glu2-B)] (1)

The PCA efficiency for each couple (Glu1 or Glu2 in N- or C-ter) was assessed in a prior experiment and the best combinations were selected further investigation.

dx.doi.org/10.17504/protocols.io.jekcjcw [PROTOCOL DOI].

### Bioluminescence resonance energy transfer (BRET)

HEK-293T cells were plated in 384 plates with 3000 cells per well in 50 μL of medium and transfected 3h later using FuGENE6 (Promega) with a total of 25 ng of plasmids expressing YFP- and Nluc-tagged proteins at a ratio of 1:1. When specified, cells were transfected with different ratios of DNA (from 1 YFP:2 Nluc to 4 YFP:1 Nluc) per condition. Direct bioluminescence from the donor (Nluc) and the acceptor (YFP, noted NlucY) was measured 48h later using the Wallac 1420 VICTOR 3V multilabel plate reader (PerkinElmer). Next, the energy transfer between the Nluc and YFP (BRET) was calculated according to the following formulas (2 and 3) and normalized to a YFP-Nluc linked recombinant protein [55].

$$CF_{value}=NlucY_{value}\left(prot\text{-}Nluc\right)/Nluc_{value}\left(prot\text{-}Nluc\right)$$ (2)

$$netBRET=[NlucY_{value}\;\text{-}\;(Nluc_{value}\;x\;CF_{value})]/Nluc_{value}$$ (3)

All combinations were assessed and combinations producing more than 0.05 of netBRET (with an N-ter tag if possible) were selected for further investigation. A threshold of specific interaction was determined using the mean+3SD (simplified 3SD) of the negative controls within each experiment.

dx.doi.org/10.17504/protocols.io.jepcjdn [PROTOCOL DOI].

### Ethics statement

All mice experiments were performed in accordance with guidelines of the European and French guidelines (Directive 86/609/CEE and Decree 87–848 of 19 October 1987) and the Institut Pasteur Safety, Animal Care and Use Committee, and approved by the French Administration (Ministère de l’Enseignement et de la Recherche) under the number O522-02. All animals were handled in strict accordance with good animal practice.

### In vivo experiments

Three-weeks old BALB/c or C57BL/6 (Charles River) were infected by intramuscular injection of 1000 focus-forming units (FFU) and monitored over 21 days. Mice were sacrificed at 9 days post infection or upon the apparition of late infection symptoms (humane end point). The infection was confirmed by RT-qPCR.

### RNA isolation, reverse transcription and quantitative real-time PCR

Total RNA was isolated using trizol. Reverse transcription was performed on 1,2 mg of RNA using Superscript II (Invitrogen) with 2 pmol of oligodT primers (Fermentas) in a final volume of 20 μL. Transcription analysis was performed on 100 ng of total RNA using Taqman Power SYBR Green (Applied Biosystems) in a 7500 instrument (Applied Biosystems) and Quantitect primers (Qiagen), following manufacturer instruction. Relative quantification was performed using GAPDH gene as endogenous control gene. Results were analyzed using 7500 SDS software v2 (Applied Biosystems).

### Statistical analysis

Multiple comparisons of data were performed by ANOVA using the GraphPad Prism software.

## Supporting information

S1 Fig Mass spectrometry data processing and analysis. A. From the raw data (n = 553), reverse proteins, potential contaminants and only identified by site proteins were filtered out, a log2 transformation was applied to the LFQ values and only the proteins identified at least more than once in a condition were kept (n = 329). Missing values of the dataset were imputed according to Tyanova et al. 2016. A total of 49 proteins are significantly identified as specific of FG-RelAp43 compared to FG. * = corresponding pValue are indicated in Fig 1A.
B. From the 49 protein identified as specific of RelAp43, LFQ ratio of [FG-RelAp43 + V5-Mtha] / [FG-RelAp43 + V5-CAT] was used to determine the effect of Mtha on the proteins purified with RelAp43.
(PDF)

S2 Fig Network of proteins identified by MS in a complex with RelAp43. Protein-protein interaction networks were determined using STRING v10 (minimum score: 0.9) and visualized with Cytoscape v3.4 based on MS hits identified after RelAp43 purification (FG-RelAp43 / FG > 0 in presence of CAT and/or M_Tha_). RelA was included in the analysis. Only the proteins determined as significant and the proteins involved in the NF-κB pathway are labeled with their gene name. A global function for each major protein cluster (n>10) was identified using DAVID v6.8 Functional annotation clustering tool. Node size is relative to the number of edges. NFKB1 = p105/p50, TNIP2 = ABIN2, MAP3K8 = TPL2, REL = cRel, NFKB2 = p100/p52, NFKBIA/B/E = IkBα/β/ε.
(PDF)

S3 Fig Alignment of the C-terminal sequence of RelAp43 and p65/RelA. C-terminal sequences of RelAp43 and p65/RelA, starting at R336 to show the last Arg and Lys present on the C-terminal part of their identical RHD sequence. Blue = homologous sequence, Red = Arg or Lys, * = localization of specific peptide of RelAp43 or RelA/p65 after an in-silico tryptic digestion.
(PDF)

S4 Fig Controls of net BRET. A. Interaction between STAT1 and p50 or with itself assessed by net BRET as described in Fig 3B (left panel). Nluc (center panel) and YFP (right panel) values were measured for each DNA ratio. Briefly, BRET and YFP signal were measured in living HEK 293T cells 48h after transfection of plasmids expressing YFP- and Nluc-tagged proteins at 5 ratios of DNA, from 1:2 to 8:1. The variations of DNA transfected is represented by a gradient of blue for the Nluc and yellow for the YFP. Each value is the mean between 3 experiments with error bars representing the standard deviation.
B. Nanoluciferase values (above) and YFP surface (below) measured across each ratios shown in Fig 3B–3E and S3A Fig.
(PDF)

S5 Fig CCL5 and CXCL10 expression is induced during RABV infection and not controlled by the M protein. A-D. Relative quantification of *CCL5* (A and C) and *CXCL10* (B and D) mRNA expression in the brain of mice infected by Tha or Th4M viruses. Six weeks old BALB/c (A and B) or C57BL/6 (C and D) were infected by intramuscular injection of 1000 UFF and monitored over 21 days. The mice were sacrificed upon the apparition of late infection symptoms and mRNA was extracted from the brain. The expression of genes was studied by RTqPCR analysis. The level of gene expression was normalized according to the level of GAPDH reporter gene in non-infected mice. Six mice were used per condition. Error bars represent the standard deviation.
(PDF)

S6 Fig Controls of mice infections prior to the experimental end point. A-D. Six weeks old BALB/c mice were mock, Tha or Th4M infected by intramuscular injection. The mice were sacrificed at 9 days post infection or at the experimental end point and mRNA was extracted from the brain. Viral RNA (A) mRNA expression was quantified at 9 days post infection or at the experimental end point. *IFNß* (B), *CXCL2* (C) and *TNF* (D) mRNA expression was quantified at 9 days post infection. Results are the mean and standard deviation obtained from 5 mice.
(PDF)

S1 Table Observed and reported interactions between the matrix proteins, RelAp43, p105/p50, ABIN2, TPL2. (DOCX)

S2 Table Representation of all recombinant proteins. Proteins are orientated from the N- to the C-terminus.
(DOCX)
