# Supplementary figures and images for: Regulation of NF-κB by the p105-ABIN2-TPL2 complex and RelAp43 during rabies virus infection
Source: PLoS Pathog. 2017 Oct 30;13(10):e1006697. doi: 10.1371/journal.ppat.1006697 (PMC5679641; doi:10.1371/journal.ppat.1006697)

A

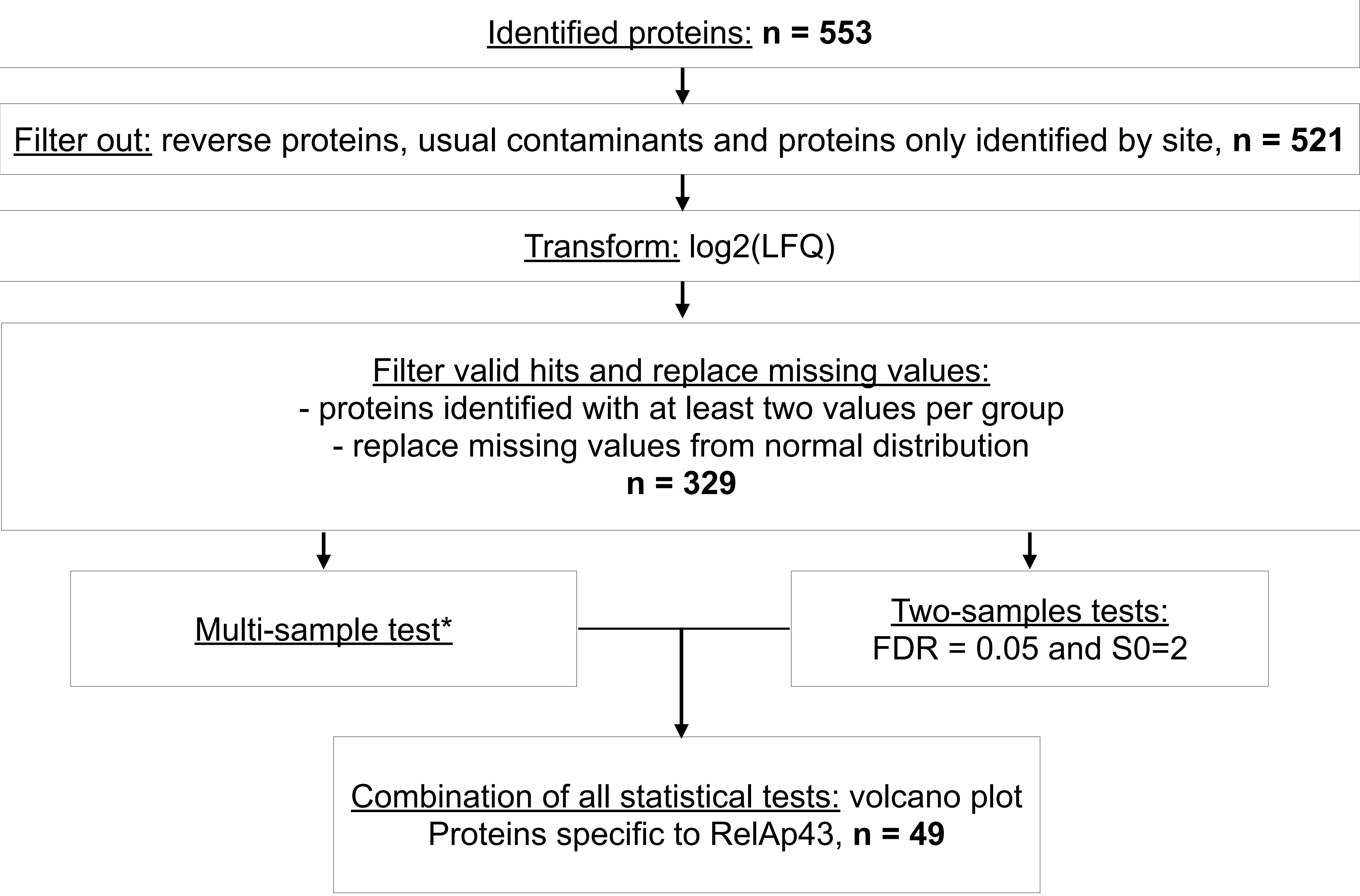

B

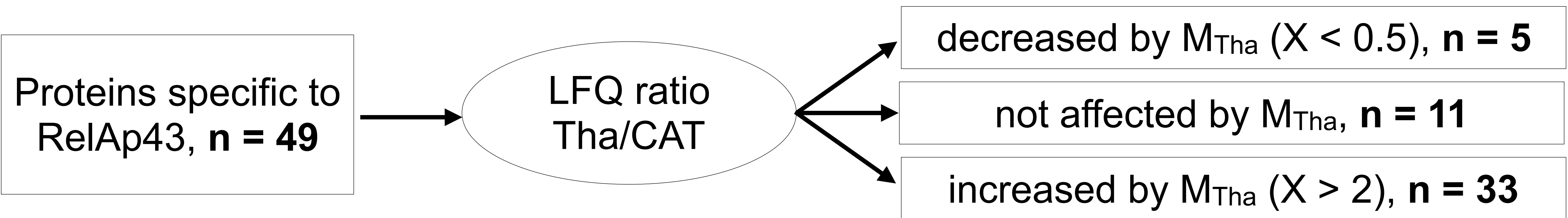

Supplement: S1 Fig — A. From the raw data (n = 553), reverse proteins, potential contaminants and only identified by site proteins were filtered out, a log2 transformation was applied to the LFQ values and only the proteins identified at least more than once in a condition were kept (n = 329). Missing values of the dataset were imputed according to Tyanova et al. 2016. A total of 49 proteins are significantly identified as specific of FG-RelAp43 compared to FG. * = corresponding pValue are indicated in Fig 1A. B. From the 49 protein identified as specific of RelAp43, LFQ ratio of [FG-RelAp43 + V5-Mtha] / [FG-RelAp43 + V5-CAT] was used to determine the effect of Mtha on the proteins purified with RelAp43. (PDF) [file ppat.1006697.s001.pdf]

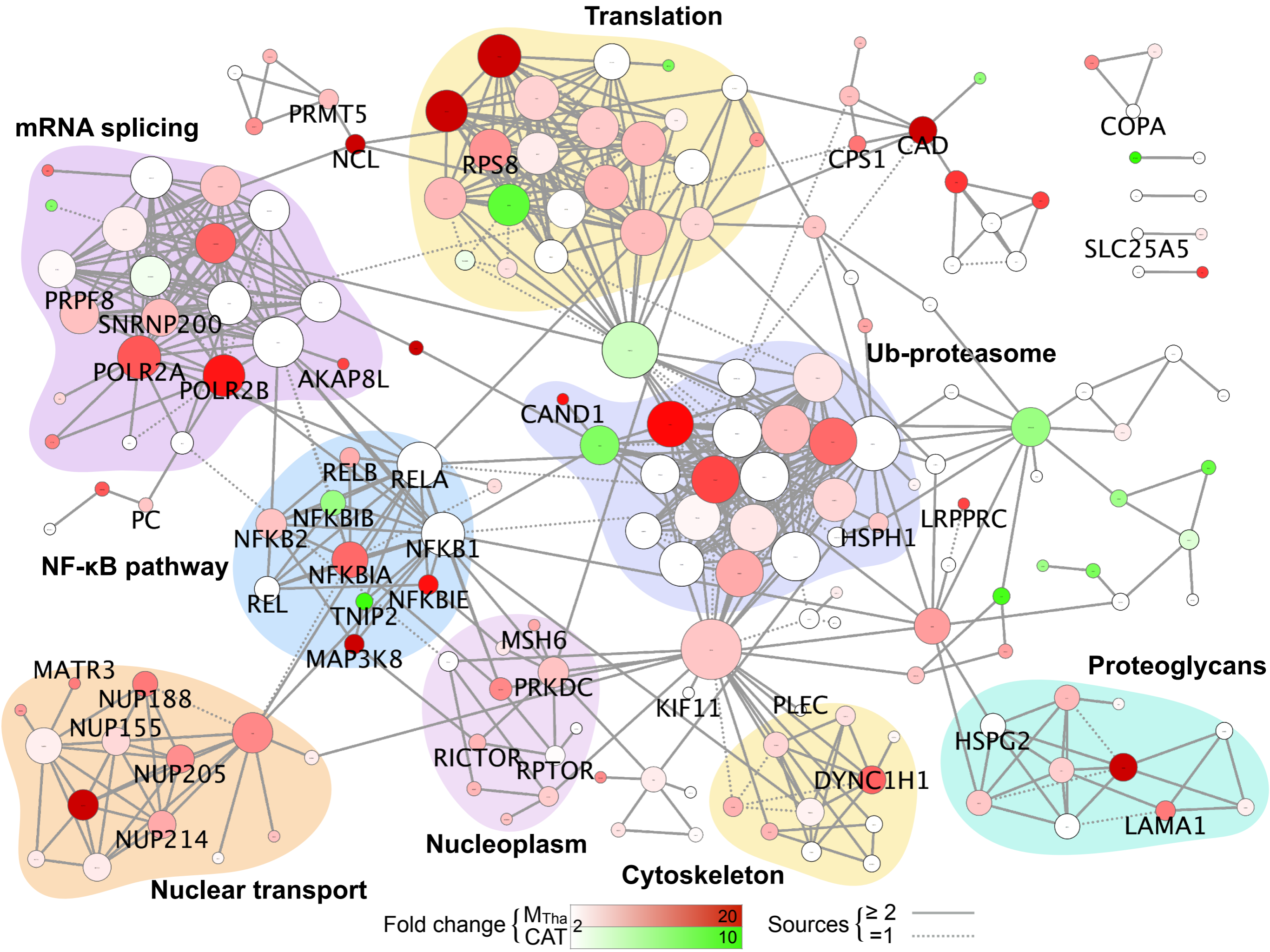

Supplement: S2 Fig — Protein-protein interaction networks were determined using STRING v10 (minimum score: 0.9) and visualized with Cytoscape v3.4 based on MS hits identified after RelAp43 purification (FG-RelAp43 / FG > 0 in presence of CAT and/or MTha). RelA was included in the analysis. Only the proteins determined as significant and the proteins involved in the NF-κB pathway are labeled with their gene name. A global function for each major protein cluster (n>10) was identified using DAVID v6.8 Functional annotation clustering tool. Node size is relative to the number of edges. NFKB1 = p105/p50, TNIP2 = ABIN2, MAP3K8 = TPL2, REL = cRel, NFKB2 = p100/p52, NFKBIA/B/E = IkBα/β/ε. (PDF) [file ppat.1006697.s002.pdf]

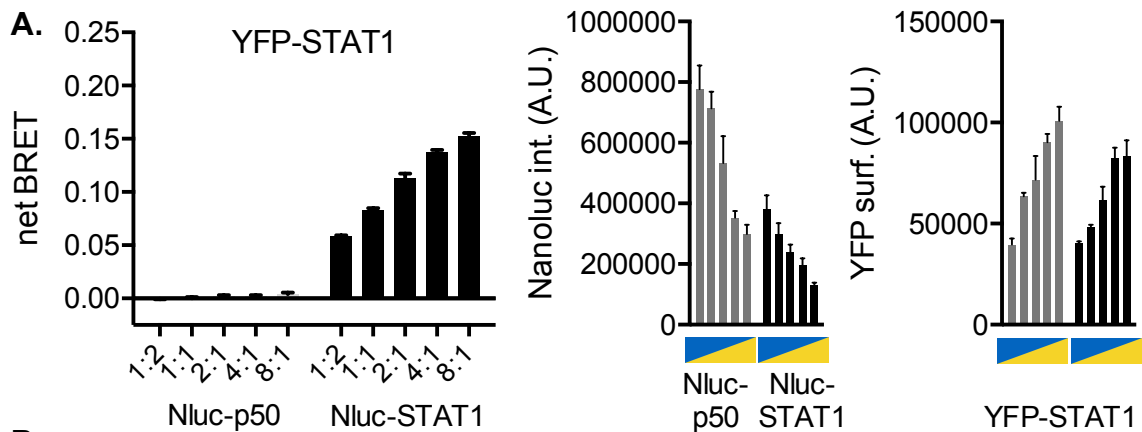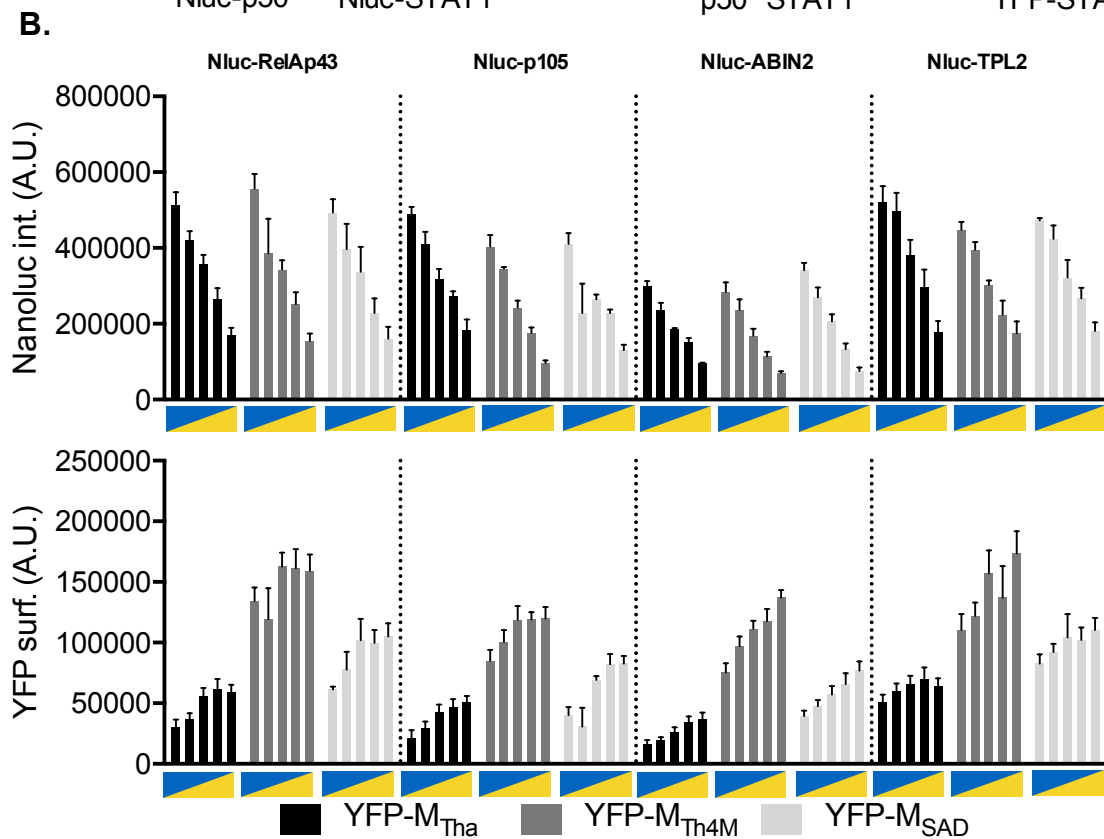

Supplement: S4 Fig — A. Interaction between STAT1 and p50 or with itself assessed by net BRET as described in Fig 3B (left panel). Nluc (center panel) and YFP (right panel) values were measured for each DNA ratio. Briefly, BRET and YFP signal were measured in living HEK 293T cells 48h after transfection of plasmids expressing YFP- and Nluc-tagged proteins at 5 ratios of DNA, from 1:2 to 8:1. The variations of DNA transfected is represented by a gradient of blue for the Nluc and yellow for the YFP. Each value is the mean between 3 experiments with error bars representing the standard deviation. B. Nanoluciferase values (above) and YFP surface (below) measured across each ratios shown in Fig 3B–3E and S3A Fig. (PDF) [file ppat.1006697.s004.pdf]

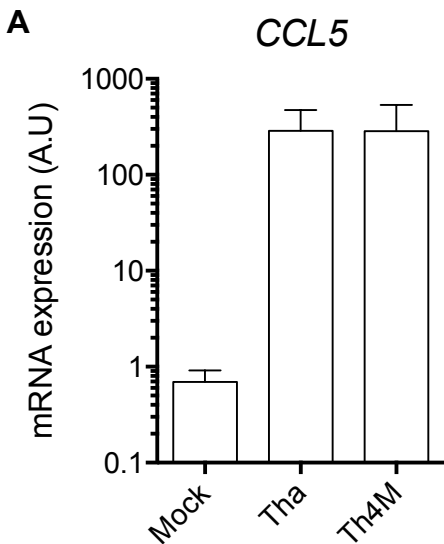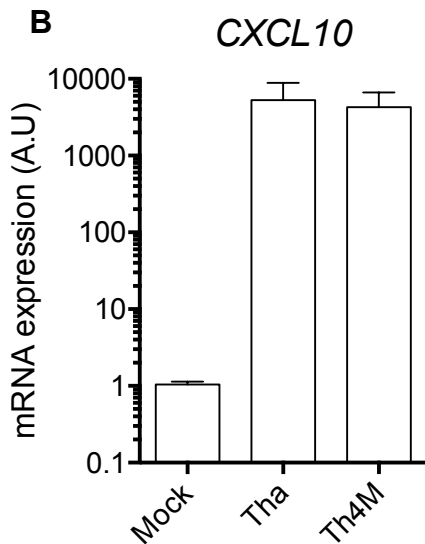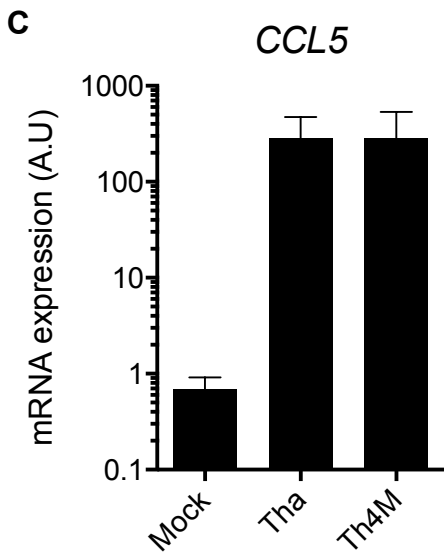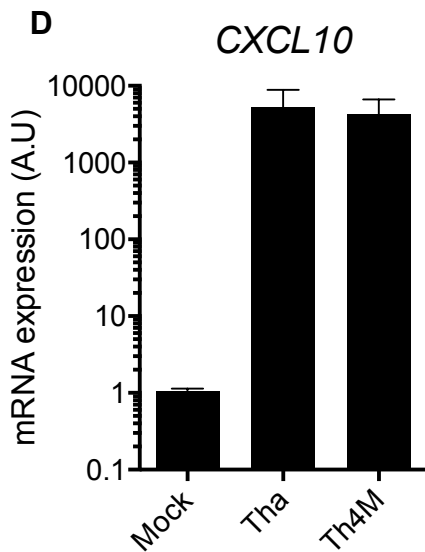

Supplement: S5 Fig — A-D. Relative quantification of CCL5 (A and C) and CXCL10 (B and D) mRNA expression in the brain of mice infected by Tha or Th4M viruses. Six weeks old BALB/c (A and B) or C57BL/6 (C and D) were infected by intramuscular injection of 1000 UFF and monitored over 21 days. The mice were sacrificed upon the apparition of late infection symptoms and mRNA was extracted from the brain. The expression of genes was studied by RTqPCR analysis. The level of gene expression was normalized according to the level of GAPDH reporter gene in non-infected mice. Six mice were used per condition. Error bars represent the standard deviation. (PDF) [file ppat.1006697.s005.pdf]

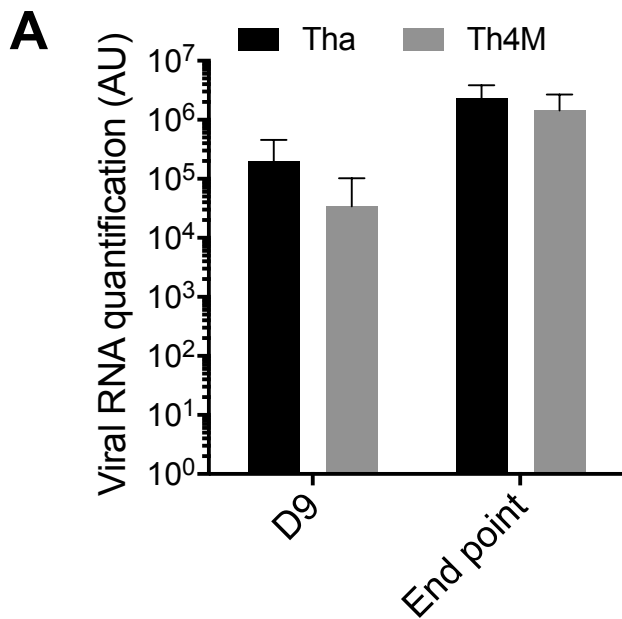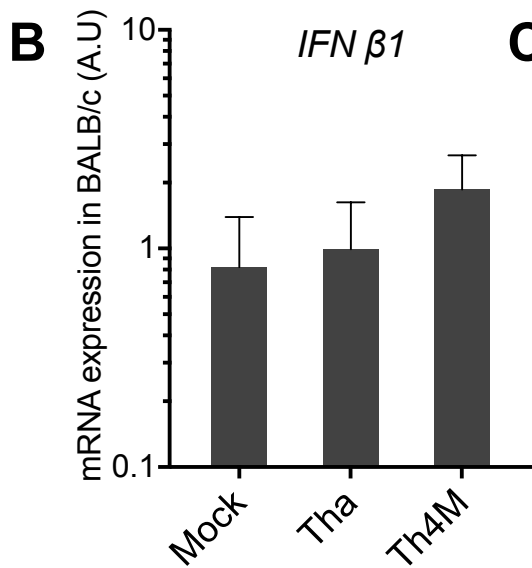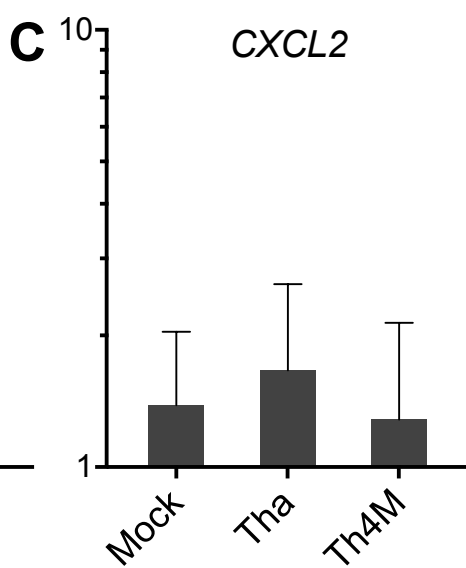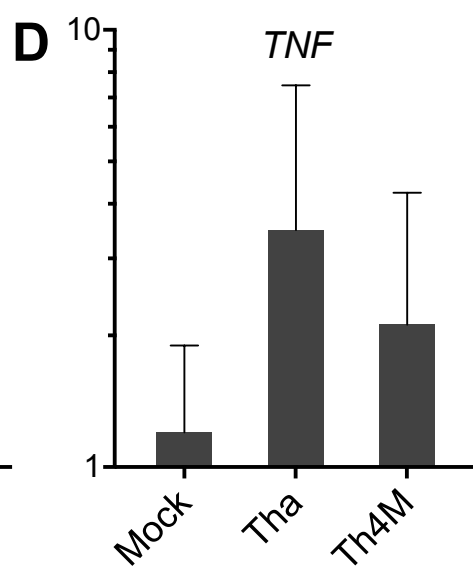

Supplement: S6 Fig — A-D. Six weeks old BALB/c mice were mock, Tha or Th4M infected by intramuscular injection. The mice were sacrificed at 9 days post infection or at the experimental end point and mRNA was extracted from the brain. Viral RNA (A) mRNA expression was quantified at 9 days post infection or at the experimental end point. IFNß (B), CXCL2 (C) and TNF (D) mRNA expression was quantified at 9 days post infection. Results are the mean and standard deviation obtained from 5 mice. (PDF) [file ppat.1006697.s006.pdf]
