# Supplementary material for: Regulation of NF-κB by the p105-ABIN2-TPL2 complex and RelAp43 during rabies virus infection
Source: PLoS Pathog. 2017 Oct 30;13(10):e1006697. doi: 10.1371/journal.ppat.1006697 (PMC5679641; doi:10.1371/journal.ppat.1006697)
Supplement: S3 Fig — C-terminal sequences of RelAp43 and p65/RelA, starting at R336 to show the last Arg and Lys present on the C-terminal part of their identical RHD sequence. Blue = homologous sequence, Red = Arg or Lys, * = localization of specific peptide of RelAp43 or RelA/p65 after an in-silico tryptic digestion. (PDF) [file ppat.1006697.s003.pdf]

RelAp43

336 - RSSASVPKPGKDFLLSHWNDRFSSVQLRSSGDEDSWAPLQTY **- 377**

RelA/p65

336 - RSSASVPKPAPQPYPFTSSLSTINYDEFPTMVFPSGQISQASALAPAPPQVLPQAPAPAPAP
AMVSALAQAPAPVPVLAPGPPQAVAPPAPKPTQAGEGTLSEALLQLQFDDEDLGALLGNSTDPAV
FTDLASVDNSEFQQLLNQGIPVAPHTTEPMLMEYPEAITRLVTGAQRPPDPAPAPLGAPGLPNGLL
SGDEDFSSIADMDFSALLSQISS **- 551**
