# Supplementary material for: Regulation of NF-κB by the p105-ABIN2-TPL2 complex and RelAp43 during rabies virus infection
Source: PLoS Pathog. 2017 Oct 30;13(10):e1006697. doi: 10.1371/journal.ppat.1006697 (PMC5679641; doi:10.1371/journal.ppat.1006697)
Supplement: S1 Table — (DOCX) [file ppat.1006697.s007.docx]

Table S1. Representation of all recombinant proteins. Proteins are orientated from the N- to the C-terminus.

|  | **TAP/MS** | **PCA** | **BRET** | |
| --- | --- | --- | --- | --- |
| **Host proteins** | FG : RelAp43 | ABIN2, p105, RelAp43, TPL2 | ABIN2, p105, p50, RelAp43, TPL2 STAT1 | |
| **Viral proteins** | V5 : M_Tha_ | / | / | M_Tha_, M_SAD_, M_Th4M_,  P (only N-ter) |
| **Other proteins** | V5 : CAT | / | / | / |
| **Tags**  **(N>C)** | 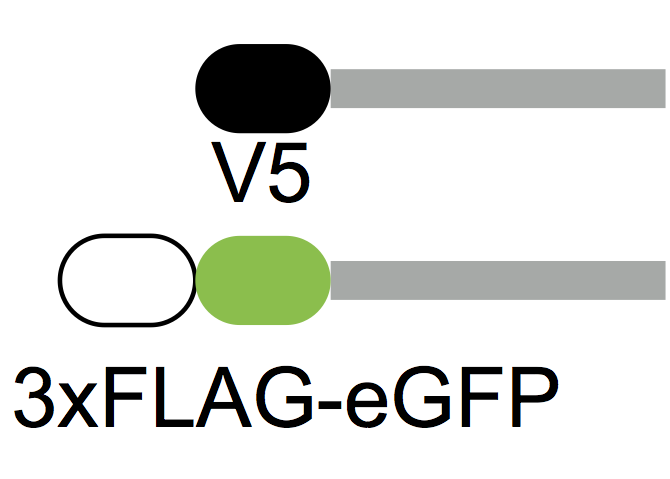 | 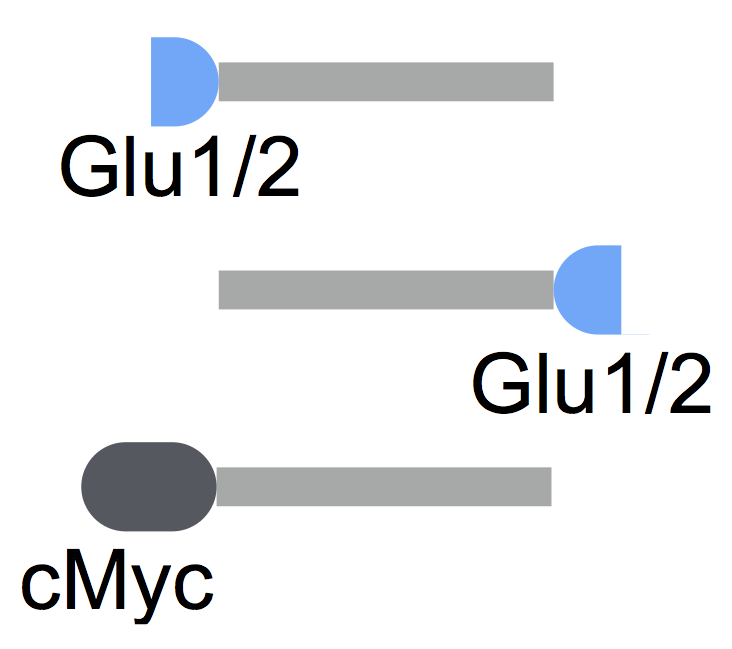 | 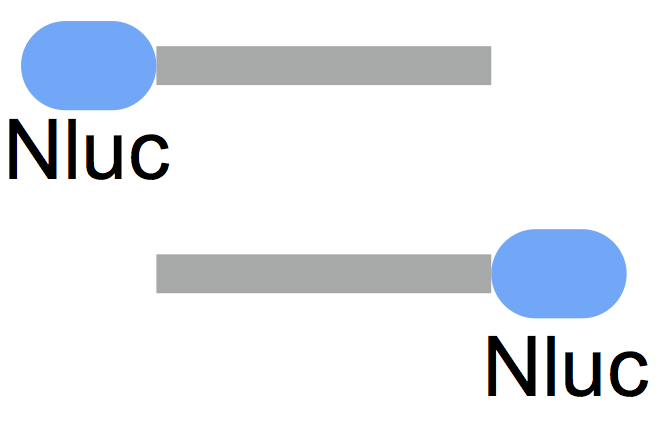 | 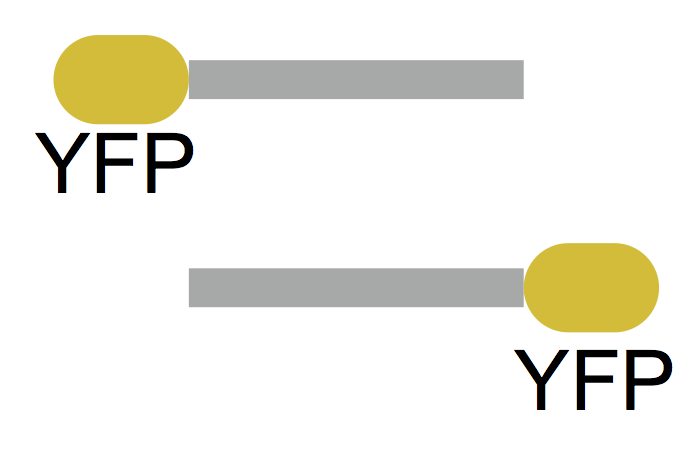 |
