# Supplementary material for: Regulation of NF-κB by the p105-ABIN2-TPL2 complex and RelAp43 during rabies virus infection
Source: PLoS Pathog. 2017 Oct 30;13(10):e1006697. doi: 10.1371/journal.ppat.1006697 (PMC5679641; doi:10.1371/journal.ppat.1006697)
Supplement: S2 Table — Proteins are orientated from the N- to the C-terminus. (DOCX) [file ppat.1006697.s008.docx]

Table S2. Observed and reported interactions between the matrix proteins, RelAp43, p105/p50, ABIN2, TPL2

|  | Literature | Our paper |
| --- | --- | --- |
| RelAp43-p105/p50 | Interacts: Luco et al., 2012 | Interacts (BRET, TAP/MS, PCA) |
| TPL2-p105/p50 | Interacts with p105: Belich et at., 1999 (Co-IPs, Interaction mapped) | Interacts with p105 only (BRET)  Enhanced by RelAp43 (PCA) |
| ABIN2-TPL2 | Interacts with TPL2: Lang et al., 2004 (Co-IPs, Interaction mapped) | Interacts (BRET)  Needs RelAp43 or p105 (PCA) |
| ABIN2-p105/p50 | Interacts with p105 through TPL2: Lang et al., 2004 (Co-IPs, Interaction mapped) | Interacts with p105 only (BRET)  Needs TPL2 (PCA) |
| ABIN2-RelAp43 | - | Interacts (BRET, TAP/MS)  Needs p105 (PCA) |
| TPL2-RelAp43 | - | Interacts (BRET, TAP/MS)  Needs p105 (PCA) |
| M-p105 | - | No interaction (BRET) |
| M-RelAp43 | M_Tha_ interacts but not M_SAD_: Luco et al., 2012 (Y2H, Co-IP), or M_Th4M_: Ben Khalifa et al., accepted (Co-IPs, Interaction mapped) | M_Tha_ interacts but not M_Th4M_ or M_SAD_ (BRET) |
| M-ABIN2 | - | Higher interaction of M_Tha_ than M_Th4M_ ou M_SAD_ (BRET) |
| M-TPL2 | - | M proteins interact (BRET) |
